# Mechanistic Modeling of Monoglyceride Lipase Covalent Modification Elucidates the Role of Leaving Group Expulsion and Discriminates Inhibitors with High and Low Potency

**DOI:** 10.1021/acs.jcim.2c00140

**Published:** 2022-05-17

**Authors:** Francesca Galvani, Laura Scalvini, Silvia Rivara, Alessio Lodola, Marco Mor

**Affiliations:** † Dipartimento di Scienze degli Alimenti e del Farmaco, Università degli Studi di Parma, Parco Area delle Scienze 27/A, I-43124 Parma, Italy; ‡ Microbiome Research Hub, University of Parma, Parco Area delle Scienze 11/A, I-43124 Parma, Italy

## Abstract

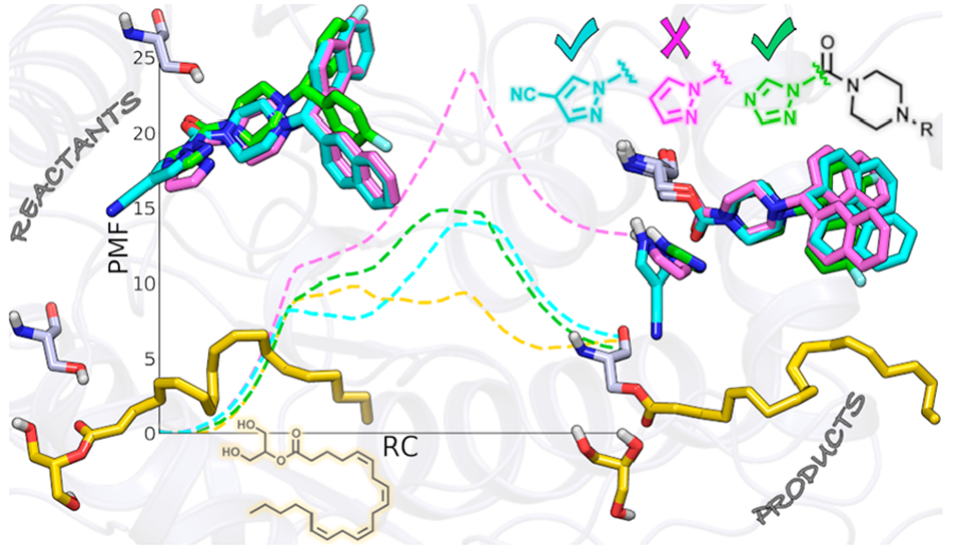

Inhibition of monoglyceride lipase (MGL), also known as monoacylglycerol lipase (MAGL), has emerged as a promising approach for treating neurological diseases. To gain useful insights in the design of agents with balanced potency and reactivity, we investigated the mechanism of MGL carbamoylation by the reference triazole urea SAR629 (IC_50_ = 0.2 nM) and two recently described inhibitors featuring a pyrazole (IC_50_ = 1800 nM) or a 4-cyanopyrazole (IC_50_ = 8 nM) leaving group (LG), using a hybrid quantum mechanics/molecular mechanics (QM/MM) approach. Opposite to what was found for substrate 2-arachidonoyl-*sn*-glycerol (2-AG), covalent modification of MGL by azole ureas is controlled by LG expulsion. Simulations indicated that changes in the electronic structure of the LG greatly affect reaction energetics with triazole and 4-cyanopyrazole inhibitors following a more accessible carbamoylation path compared to the unsubstituted pyrazole derivative. The computational protocol provided reaction barriers able to discriminate between MGL inhibitors with different potencies. These results highlight how QM/MM simulations can contribute to elucidating structure–activity relationships and provide insights for the design of covalent inhibitors.

## Introduction

**Figure 1:**
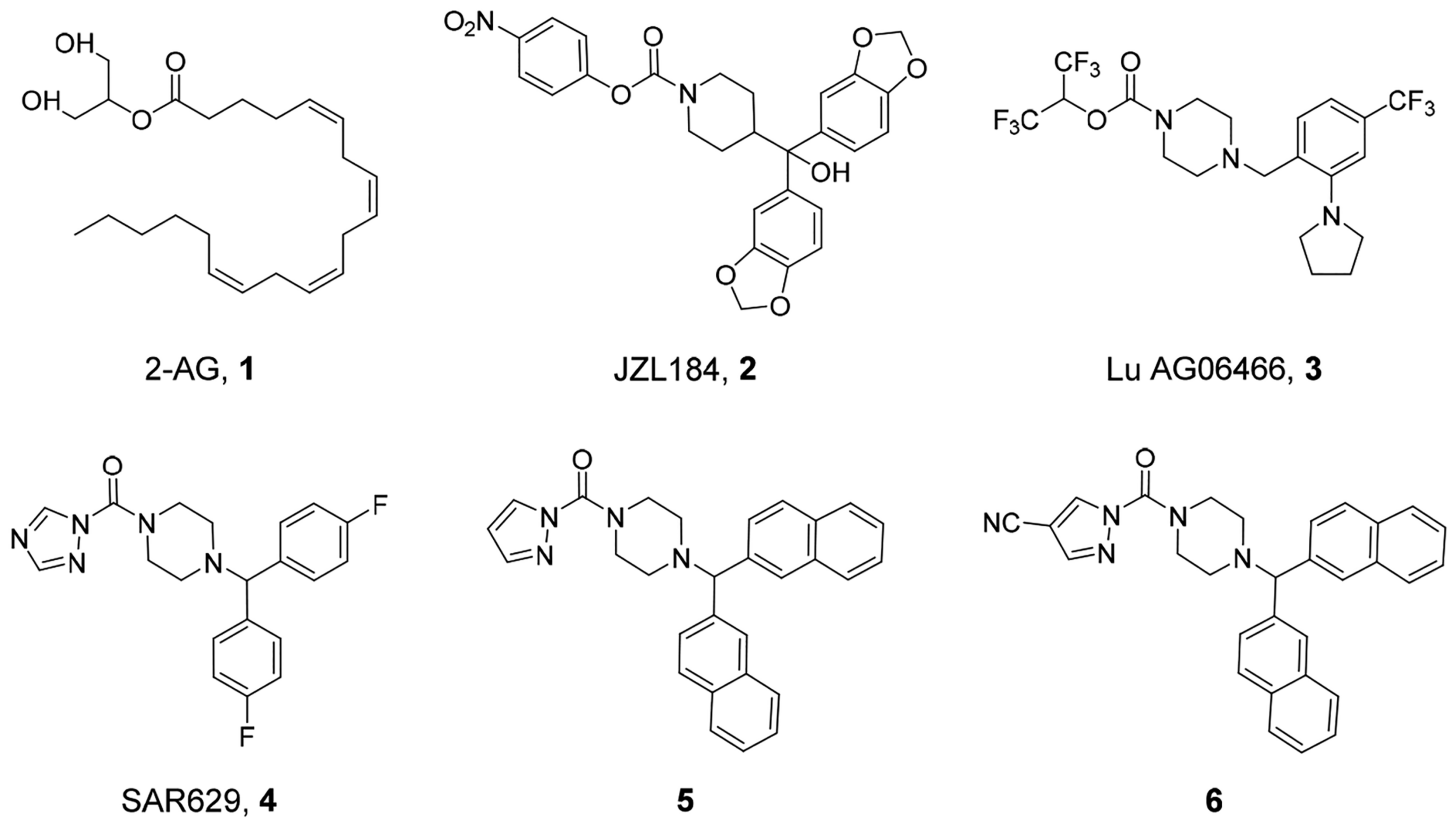
Chemical structures of 2-AG (**1**) and selected inhibitors (**2**–**6**).

Monoglyceride lipase (MGL), also named monoacylglycerol lipase (MAGL), is a serine hydrolase that catalyzes the hydrolytic deactivation of monoacylglycerols into glycerol and fatty acids, with a marked preference for the ester derivatives of arachidonic acid.^1^ MGL is widely expressed in the mammalian body, including the central nervous system (CNS), where it is localized to presynaptic nerve terminals. MGL contributes to the deactivating cleavage of the most abundant endocannabinoid in the brain, 2-arachidonoyl-*sn*-glycerol (2-AG, compound **1**, Figure 1). Similar to the other endocannabinoid anandamide, **1** is released upon demand by postsynaptic neurons and exerts a wide array of effects,^2^ including antinociception^3^ and neuroprotection.^4^

**Figure 2:**
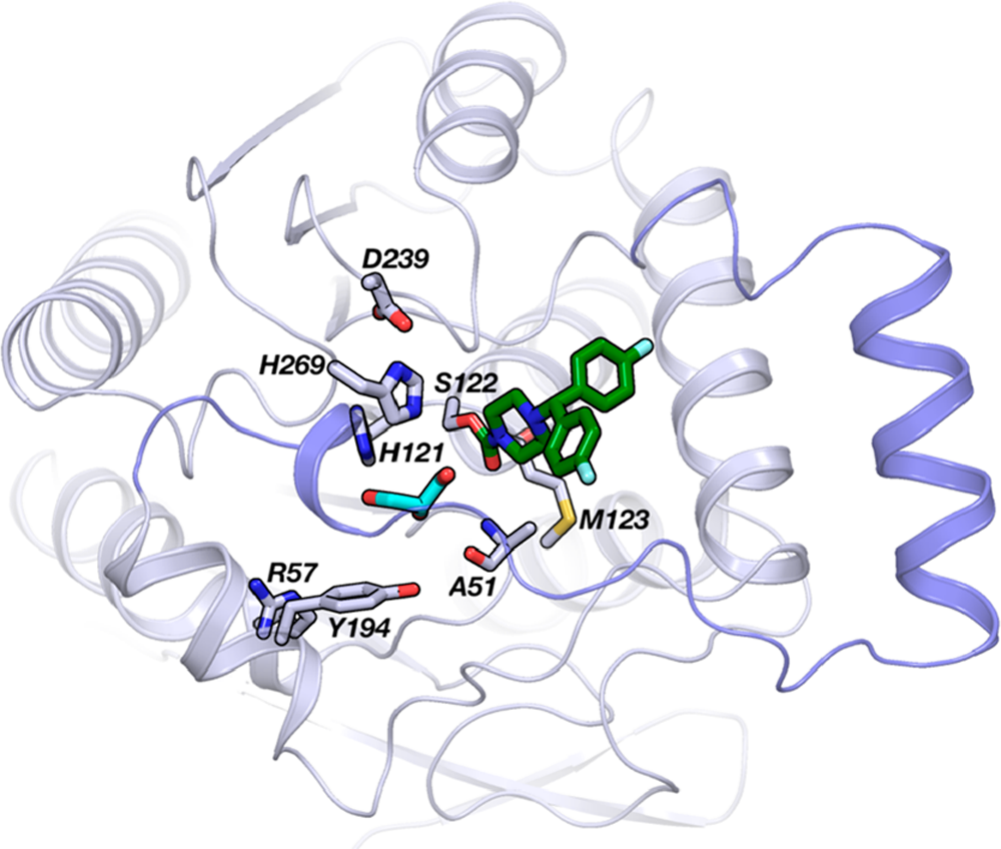
X-ray structure of human MGL covalently bound to SAR629 (compound **4**, in Figure 1, PDB code: 3JWE, chain B). MGL carbon atoms are colored in gray and SAR629 in dark green. The secondary structure of MGL is represented with gray cartoon, and the lid domain in its open conformation is blue-colored. A glycerol molecule, occupying a hydrophilic pocket of the enzyme and taken from the X-ray structure 6AX1 (chain A) superimposed to 3JWE, is also represented in cyan carbon atoms.

MGL belongs to the α/β hydrolase superfamily and shares with the other members of this group a conserved β-sheet core hosting the Ser122–His269–Asp239 catalytic triad.^5^ The core region of the enzyme is covered by a flexible lid domain that serves as an anchoring interface for the cellular membrane, where it controls the recruitment of substrates and the release of the products.^6^ The catalytic triad of MGL is located at the bottom of a channel delimited by the lid domain (Figure 2). The side chains of the triad residues are involved in a hydrogen-bond (H-bond) network that enhances the nucleophilic character of the nucleophile Ser122.^7−9^ Close to Ser122, the NH backbone groups of Ala51 and Met123 form the oxyanion hole, which is believed to stabilize the tetrahedral intermediate (TI) generated during 2-AG hydrolysis. Beyond the oxyanion hole, the binding channel terminates with a cleft, lined by a set of polar residues that include Arg57, His121, and Tyr194 and connected by a narrow opening to the solvent.

In recent years, the search for potent inhibitors of MGL has led to the identification of different classes of compounds,^10,11^ ranging from allosteric cysteine-targeting modulators^12^ to orthosteric inhibitors, comprising both covalent agents (i.e., carbamates^13^ and tertiary ureas^14^) and noncovalent inhibitors.^15^ Covalent inhibitors of MGL include JZL184 (compound **2**, Figure 1)^16^ reported as the first agent able to carbamoylate Ser122, and the first-in-class inhibitor Lu AG06466/ABX-1431 (compound **3**, Figure 1),^17^ which recently completed a phase 2 clinical trial for the treatment of Tourette’s syndrome^18^ and is currently being evaluated for multiple sclerosis.^19^

Beside carbamates, azole ureas represent another relevant class of covalent modifiers of MGL. This chemotype has been extensively exploited in the design of serine hydrolase inhibitors and owes its inhibitory activity to the presence of a tertiary urea incorporating an azole ring that can act as leaving group (LG).^20^ A prototypical example of this class is represented by the piperazine triazole urea SAR629 (compound **4**, Figure 1), which potently inhibits mouse MGL (IC_50_ = 0.2 nM) in an activity-based protein profiling (ABPP) assay.^21^ X-ray data show that this compound reacts with Ser122 forming a stable carbamoylated adduct, responsible for the irreversible inactivation of the enzyme (Figure 2).^14^ An interesting property of azole ureas is that their reactivity versus nucleophiles depends on the chemical nature of the azole ring, with tetrazoles being highly reactive,^22^ followed by triazoles, imidazoles, and pyrazoles.^23^ This trend, to some extent, can be explained by the ability of the azole ring to serve as the LG in carbamoylation reactions, with the p*K*_a_ of the azole conjugate acid used as a coarse predictor of reactivity. Within this class, pyrazole derivatives are often neglected as they display low reactivity toward nucleophiles due to their poor ability to be expelled as anionic LG in acyl transfer reactions. In certain conditions, which include activation by protonation of their basic nitrogen and expulsion of a neutral LG, pyrazole ureas may act as covalent inhibitors of serine hydrolases.^24^ This could be the case for pyrazole ureas **5** and **6** (Figure 1) that, tested in an ABPP assay similar to the one employed for **4**, were reported to inhibit mouse MGL in the micromolar (**5**, IC_50_ = 1800 nM) and nanomolar (**6**, IC_50_ = 8 nM) range, respectively.^24^ However, the mechanism of action of these pyrazole ureas still remains elusive as the introduction of an electron-withdrawing substituent (e.g., compound **6**) is expected to increase the ability of the pyrazole to act as a (anionic) LG and not to be activated by protonation within the enzyme active site.

Modulation of compound reactivity by substituents on MGL inhibition can be elucidated by hybrid quantum mechanics/molecular mechanics (QM/MM) simulations,^25,26^ which allow investigation of processes involving breaking and forming of covalent bonds,^27^ including reactions catalyzed by enzymes,^28−30^ and give key information for the rationalization of structure–reactivity relationship data^31−33^ and the design of novel compounds.^34,35^

Starting from the X-ray structure of human MGL (hMGL) covalently modified by **4**,^14^ we applied a QM/MM approach, based on umbrella sampling (US)^36^ and on DFTB3/AMBER potential,^37^ to reconstruct the potential of mean of force (PMF)^38^ of MGL acylation by the substrate 2-AG (**1**) and carbamoylation by SAR629 (**4**). This allowed us to assess the ability of QM/MM simulations to provide mechanistic insights on acylation and carbamoylation consistent with mutagenesis and structural data. The QM/MM protocol was then applied to estimate the PMF of MGL covalent modification by pyrazoles **5** and **6**, to assess the likelihood of Ser122 carbamoylation by the two compounds, and to propose a mechanistic explanation accounting for their different potency displayed in the MGL inhibition assay. Finally, to test from a drug design perspective the multiscale protocol here described, the PMF of MGL carbamoylation by a new piperazine pyrazole urea, recently reported in a granted patent, was computed and compared to that of reference inhibitors **4**–**6**.

## Results

### Molecular Model of MGL–**1** Michaelis Complex

**Figure 3:**
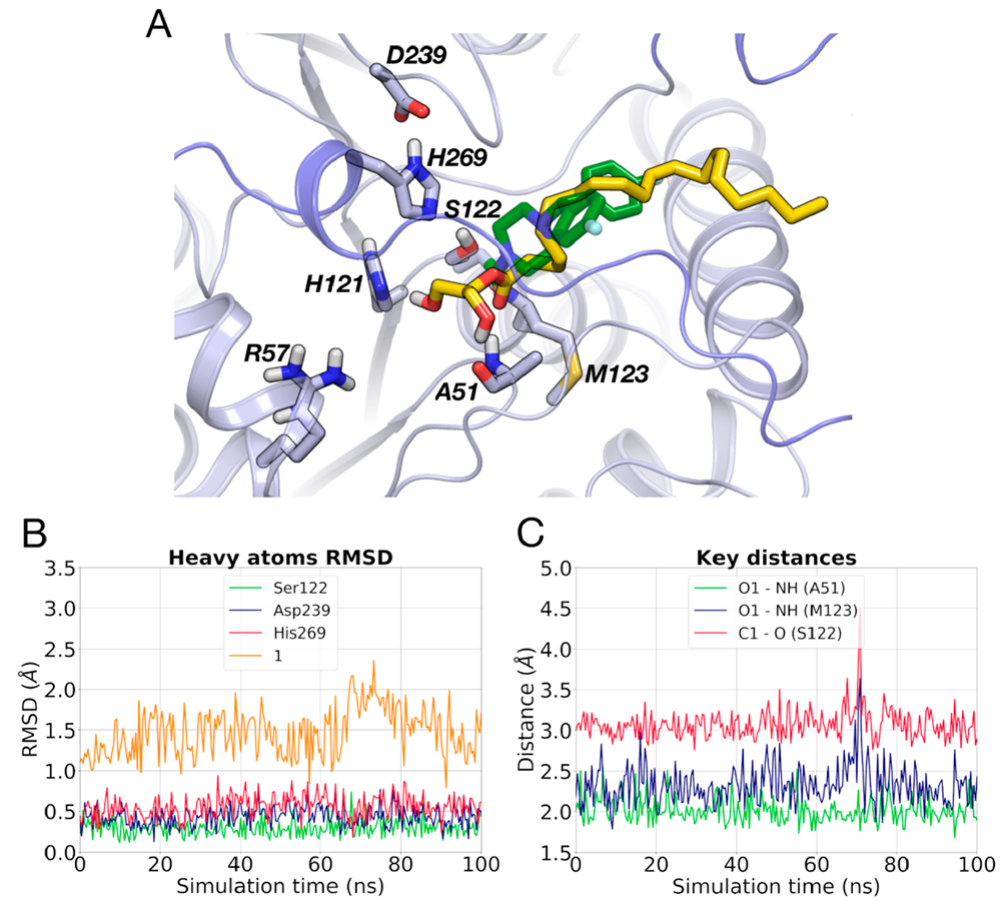
(A) Superposition of the docking pose of substrate **1** (yellow carbon atoms) and the X-ray structure of **4** (dark green carbon atoms) bound to MGL (PDB code 3JWE). Residues involved in the recognition and hydrolysis of the substrate are represented. The secondary structure of MGL is displayed in gray cartoon, and the flexible region of the lid domain is highlighted in blue. (B) RMSD analysis for the heavy atoms of the catalytic residues and of **1** during a MD simulation. (C) Interatomic distances between the carbonyl oxygen of **1** (O1) and the NH groups of Ala51 and Met123 and between the carbonyl carbon of **1** (C1) and the nucleophilic oxygen of Ser122, recorded during a MD simulation.

A key condition to investigate enzyme-catalyzed reactions is to generate a reliable model of the reactants. A three-dimensional model of the substrate–enzyme Michaelis complex was built by docking **1** (2-AG) within the active site of MGL after the removal of the cocrystallized inhibitor **4** (see Methods for details). Docking simulations identified a binding mode compatible with acylation (Figure 3A) in which the polar head of **1** occupies the catalytic site close to the nucleophile residue Ser122, with the carbonyl oxygen forming two H-bonds with the oxyanion hole residues and the glycerol establishing polar interactions with the His121 side chain and Ala51 backbone. In this model, the terminal hydroxyl groups of **1** occupy a position close to glycerol molecules cocrystallized in some X-ray structures of MGL (PDB codes 6AX1(39) and 3HJU(5)). The acyl chain of **1** is located in a cavity defined by the lid domain in its open conformation, where also the lipophilic portions of available cocrystallized inhibitors are accommodated.^40^

To evaluate its dynamic stability, the MGL–**1** system was submitted to a 100 ns long molecular dynamics (MD) simulation using AMBER force field.^41^ In this simulation, the catalytic triad maintains an arrangement close to the starting structure showing a root-mean-square deviation (RMSD) lower than 1.0 Å for each residue (Figure 3B). Substrate **1** is characterized by a slightly higher mobility (RMSD = 1.47 ± 0.29 Å), due to a slight rearrangement of the acyl chain, driven by the flexibility of its first three covalent bonds (described by τ1, τ2, and τ3 dihedral angles in Figure S1 in the Supporting Information). In contrast, the glycerol moiety shows limited rearrangement and remains close to the conformation observed in the docking pose (Figure S1). Analysis of the H-bonds formed by the catalytic triad or by **1** within the MGL active site shows that His269 can simultaneously accept and donate a H-bond from Ser122 and to Asp239, respectively (Figure S1), with **1** maintaining all of the interactions observed in the docking pose (Figures 3C and S1). Overall, these interactions keep the carbonyl carbon of **1** at a distance of 3.07 ± 0.18 Å from the oxygen atom of the nucleophile Ser122 (Figure 3C), consistent with an incoming nucleophilic attack. Similar results were obtained in two other MD replicas.

### Catalytic Mechanism for MGL Acylation by **1**

**Figure 4:**
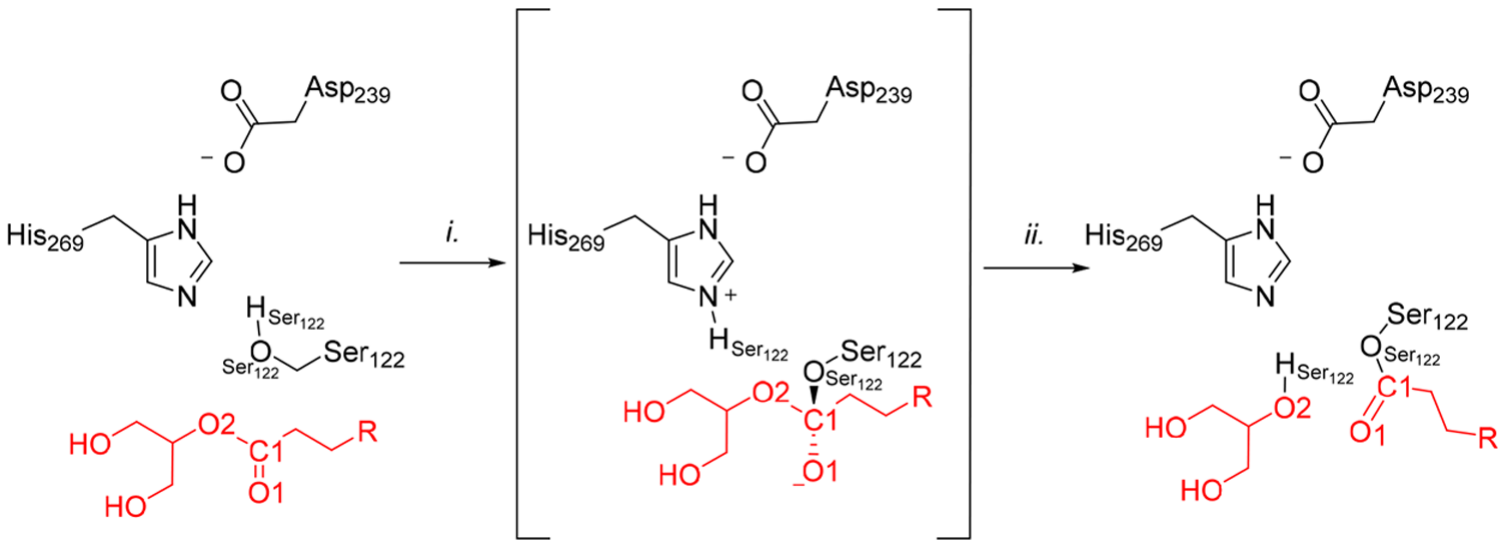
Mechanism of MGL acylation by **1**: (i) TI formation through nucleophilic attack by Ser122 to the carbonyl carbon (C1) of **1**; (ii) TI decomposition prompted by LG (glycerol) expulsion.

Acylation of MGL by **1** (2-AG) is expected to occur with a catalytic mechanism similar to the one shared by other esterases, including lipases.^42^ This includes a first step in which the nucleophilic serine attacks the carbonyl carbon of **1**, generating a tetrahedral intermediate (TI), and a second step in which the TI collapses, due to the expulsion of the glycerol LG, with the consequent formation of the acylenzyme (Figure 4).

The Michaelis complex equilibrated by classic MD was submitted to a QM/MM MD (200 ps), applying a DFTB3/AMBER potential.^37^ Atoms treated at QM level include side chains of the catalytic triad Ser122–His269–Asp239 and the 1,3-dihydroxypropan-2-yl propionate fragment of **1** (see Methods for details). The resulting structure was employed to simulate MGL acylation. The reaction was modeled converting the Michaelis complex into the acylenzyme by restraining the system along a reaction coordinate (RC) defined as the difference of two key distances, the first accounting for the nucleophilic attack [*d*(O_Ser122_, C1)] and the second for the expulsion of the LG [*d*(C1, O2)].

A first guess path for MGL acylation was obtained forcing the progression of the system along the RC by steered-MD (SMD).^43^ Analysis of the reaction trajectory shows that acylation occurs through a series of consecutive events: (i) activation of the nucleophile, (ii) nucleophilic attack with generation of a TI, and (iii) protonation of the LG followed by its expulsion, with formation of the acylenzyme. While the SMD simulation provided a feasible reaction pathway for MGL acylation, this method can only coarsely estimate the reaction energetics of a transformation (Figure S2), due to the nonequilibrium conditions applied.^43^ We thus extracted a set of geometries along the reaction path covering the space of the RC connecting reactants and products and used them as starting points for US simulations (see Methods for details).^36^ This sampling approach, in combination with the weighted histogram analysis method (WHAM),^44,45^ allowed us to reconstruct the change in free energy due to the progression along the RC,^36,38^ also known as PMF.

**Figure 5:**
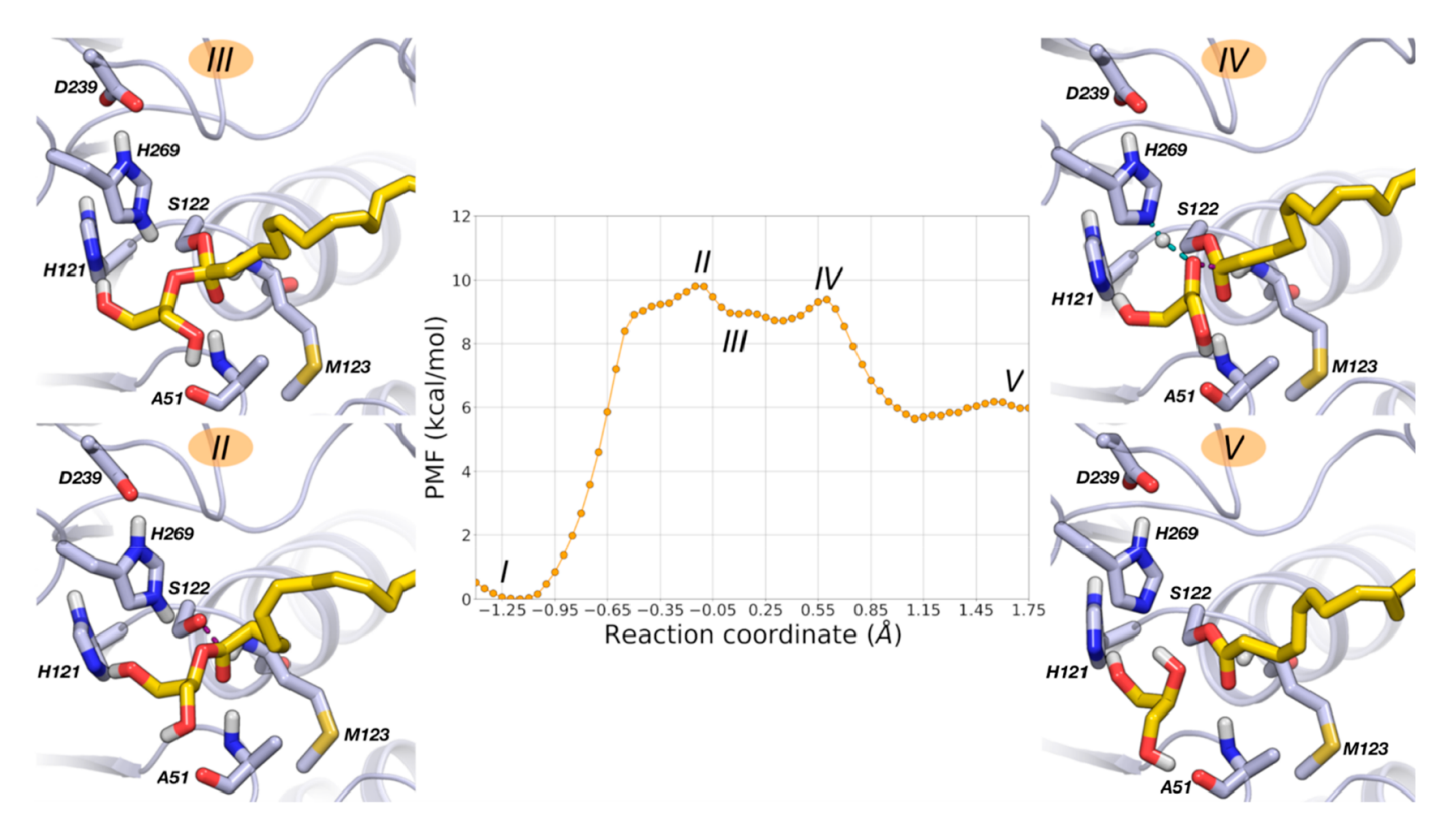
PMF for MGL acylation calculated at DFTB3/AMBER level (500 ps of simulation for each US window). Free-energy values are given in kcal·mol^–1^, and the RC is given in Å. Relevant configurations along the reaction pathway are represented: (II) TS1 (RC = −0.125 Å); (III) TI (RC = 0.10 Å); (IV) TS2 (RC = 0.55 Å); (V) acylenzyme with the expelled glycerol molecule (RC = 1.75 Å). **1** is represented with yellow carbon atoms and MGL with gray carbon atoms.

The PMF of MGL acylation (after 500 ps of US simulation per window) is reported in Figure 5. The evolution of the free energy profiles at different simulation times shows that convergence of the free energy is achieved after 400 ps of US simulation for each window (Figure S3). In this condition, the uncertainty of the reported PMF is lower than 0.2 kcal·mol^–1^.

Starting from the Michaelis complex (I on the PMF, Figure 5), acylation initiates with the deprotonation of the catalytic Ser122 by His269, which leads to an alcoholate/imidazolium pair. The cationic form of His269 is well stabilized by the negatively charged residue Asp239, which accepts a short H-bond from the imidazolium N_π_H group. Deprotonation of Ser122 triggers the nucleophilic attack to the carbonyl carbon of **1**, which takes place overcoming a free energy barrier of 10 kcal·mol^–1^ and leading to a first transition state (TS1) of the reaction (RC = −0.125 Å, configuration II, Figure 5). Analysis of geometries at TS1 (Table 1) indicates that when this configuration is reached, the proton transfer involving Ser122 and His269 is already completed, the N_His269_–H_Ser122_ distance being the same as that of the TI (1.05 ± 0.03 Å for both TS1 and TI), while the nucleophilic attack is still occurring, with O_Ser122_–C1 distance (1.61 ± 0.04 Å) higher than that observed in the TI (1.48 ± 0.04 Å). The evolution of this first step of acylation can be described following the elongation of the carbonyl bond of **1** (Table 1) and analyzing the Mulliken charges of key QM atoms (Table 2). The C1–O1 distance passes from 1.25 ± 0.02 Å in I to a greater value of 1.33 ± 0.03 Å in the TI (configuration III, Figure 5). A significant variation in the magnitude of the charges of C1 and O1 atoms is also observed, as these change from 0.76 ± 0.01 (C1) and −0.74 ± 0.03 (O1) observed in I to 0.96 ± 0.02 (C1) and −1.08 ± 0.04 (O1) in III. The TI emerges as a metastable state, as its energy becomes lower than that of TS1 by 1 kcal·mol^–1^. Nevertheless, the TI appears well stabilized by the oxyanion hole, similarly to what has been reported for other serine hydrolases.^46^ The transient character of the TI is confirmed by the second step of the acylation process, which required ∼1 kcal·mol^–1^ to occur (RC = 0.55 Å, TS2, configuration IV, Figure 5). Analysis of TS2 structure shows that protonation and expulsion of glycerol are concerted. The transfer of H_Ser122_ to the glycerol oxygen O2 is occurring (O2–H_Ser122_ distance 1.55 ± 0.24 Å) during the breakage of the C1–O2 bond, the distance between these two atoms being 1.93 ± 0.06 Å. The breakage of this bond at TS2 is evidenced by the increase in the magnitude of the negative charge on the glycerol atom O2 (−0.77 ± 0.08) compared to that of the TI (−0.62 ± 0.02). Once expulsion of glycerol is completed, the product of the acylation is formed (configuration V, Figure 5) and the C1–O1 bond assumes a length of 1.26 ± 0.02 Å, consistent with that of a carbonyl group. This final configuration shows the free glycerol occupying the same region as the glycerol in two X-ray structures of MGL (PDB codes 6AX1(39) and 3HJU,^5^Figure S4). The acylenzyme is characterized by an energy value of nearly 6 kcal·mol^–1^ above the reactants (I). The free energy of configuration V is expected to be further lowered by the removal of the glycerol from the MGL active site through the polar channel oriented versus the solvent bulk.

**Table 1:** Distances^a^ between Key Atoms Involved in MGL Acylation by **1**

	O_Ser122_–C1	C1–O2	C1–O1	O_Ser122_–H_Ser122_	N_His269_–H_Ser122_	O_Asp239_–H_His269_	O2–H_Ser122_
Michaelis complex (I)	2.58 ± 0.06	1.33 ± 0.03	1.25 ± 0.02	0.99 ± 0.03	2.02 ± 0.27	1.87 ± 0.11	3.13 ± 0.21
TS1 (II)	1.61 ± 0.05	1.47 ± 0.05	1.32 ± 0.03	1.91 ± 0.19	1.05 ± 0.03	1.74 ± 0.11	2.43 ± 0.27
TI (III)	1.48 ± 0.04	1.58 ± 0.05	1.33 ± 0.03	2.47 ± 0.29	1.05 ± 0.03	1.70 ± 0.12	1.93 ± 0.19
TS2 (IV)	1.39 ± 0.03	1.93 ± 0.06	1.28 ± 0.02	2.68 ± 0.22	1.17 ± 0.24	1.78 ± 0.12	1.55 ± 0.24
acylenzyme (V)	1.33 ± 0.03	3.09 ± 0.06	1.26 ± 0.02	3.45 ± 0.25	2.75 ± 0.42	1.92 ± 0.13	0.98 ± 0.03

a Reported as average ± SD in Å.

**Table 2:** Charges^a^ for Key Atoms Involved in MGL Acylation by **1**

	C1	O1	O2	O_Ser122_	N_His269_
Michaelis complex (I)	0.76 ± 0.01	–0.74 ± 0.03	–0.34 ± 0.02	–0.61 ± 0.02	–0.43 ± 0.02
TS1 (II)	0.96 ± 0.02	–1.05 ± 0.04	–0.54 ± 0.02	–0.62 ± 0.02	–0.04 ± 0.02
TI (III)	0.96 ± 0.02	–1.08 ± 0.04	–0.62 ± 0.02	–0.53 ± 0.02	–0.04 ± 0.02
TS2 (IV)	0.88 ± 0.03	–0.91 ± 0.05	–0.77 ± 0.08	–0.44 ± 0.02	–0.12 ± 0.12
acylenzyme (V)	0.74 ± 0.01	–0.74 ± 0.03	–0.66 ± 0.02	–0.31 ± 0.03	–0.42 ± 0.02

a Reported as average ± SD in atomic unit (au).

### Molecular Model of MGL–**4** Michaelis Complex

**Figure 6:**
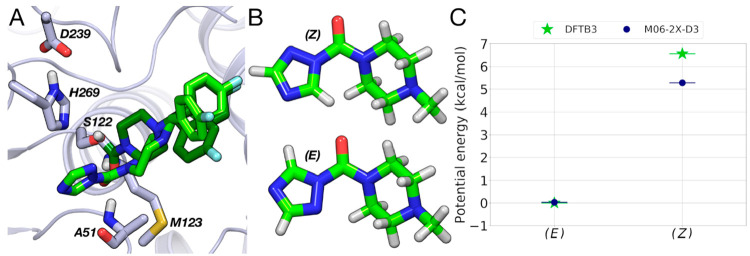
(A) Superposition of binding mode of **4** in the (*E*)-configuration (light green carbon atoms) obtained by docking with the X-ray structure of **4** bound to MGL (dark green and gray carbon atoms, respectively). (B) Piperazine triazole urea fragments modeled in (*E*)- and (*Z*)-configurations. (C) Gas-phase energies for the two piperazine triazole urea fragments calculated at DFTB3 and M06-2X-D3 levels.

A similar multiscale protocol was applied to elucidate the mechanism of inhibition of triazole piperazine urea **4**, using the X-ray structure of MGL carbamoylated by this compound. The Michaelis complex was built by docking **4** into the active site of MGL. Top-ranked docking poses show that **4** accommodates its Y-shaped portion into the lipophilic tunnel, overlapping the coordinates of the covalent fragment in the X-ray structure 3JWE.^14^ The piperazine ring of the inhibitor becomes rotated 90° with respect to the coordinates of the covalently bound molecule of **4** in 3JWE (Figure 6A) to preserve the planarity of the urea group.

Compound **4** places the urea oxygen within the oxyanion hole and triazole LG in the same pocket occupied by the glycerol moiety of **1**. Docking simulations provided two alternative poses (with similar score) for the triazole ring corresponding to (*E*)- or (*Z*)-configuration of the urea group. To identify the preferred geometry for inhibitor **4**, the gas-phase energy for a piperazine triazole urea fragment, modeled in both (*E*)- and (*Z*)*-*configurations (Figure 6B), was calculated with two different QM methods (DFTB3^47^ and M06-2X-D3^48^ with cc-PVDZ basis set). Calculations indicate that the (*E*)-configuration is preferred by several kcal·mol^–1^ regardless the level of theory applied (Figure 6C and Table S1 in the Supporting Information), which may be explained by repulsive interactions between the lone pair electrons of the N2 and O atoms in the (*Z*)-configuration.^49^

**Figure 7:**
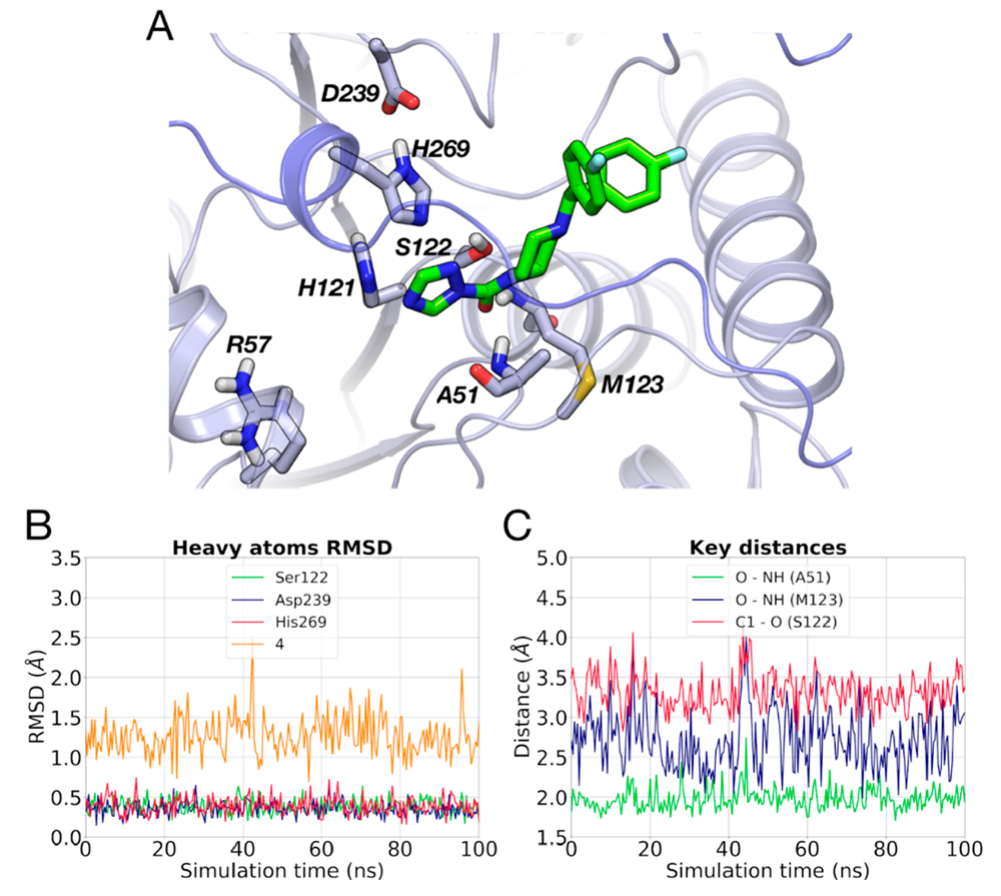
(A) Docking pose of **4** (light green carbon atoms) within MGL (gray carbon atoms). Residues involved in the recognition of the inhibitor are represented. The secondary structure of MGL is displayed in gray cartoon, and the flexible region of the lid domain is highlighted in blue. (B) RMSD analysis for the heavy atoms of the catalytic triad and of **4** during a MD simulation. (C) Interatomic distances between the carbonyl oxygen (O) of **4** and the NH groups of Ala51 and Met123 and between carbonyl carbon (C1) of **4** and the nucleophilic oxygen of Ser122, recorded during a MD simulation.

The Michaelis complex of MGL with **4** in (*E*)-configuration was submitted to a 100 ns long MD simulation using AMBER force field (Figure 7A).^41^ In this MD simulation, the catalytic triad maintains an arrangement close to the starting structure showing RMSD lower than 1.0 Å (Figure 7B). Inhibitor **4** displays limited flexibility (RMSD = 1.26 ± 0.24 Å), as this piperazine triazole urea oscillates around the docking pose. The simulation confirms that the catalytic triad forms stable interactions with His269 accepting and donating a H-bond from Ser122 and to Asp239, respectively (Figure S5). Moreover, **4** undertakes stable interactions within MGL as its carbonylic oxygen forms two H-bonds with the oxyanion hole (Figure 7C), which allow it to maintain the carbonyl carbon at 3.33 ± 0.23 Å from the Ser122 nucleophilic oxygen (Figure 7C). Similar results were obtained in two other MD replicas of the MGL–**4** complex.

### Catalytic Mechanism for MGL Carbamoylation by **4**

**Figure 8:**
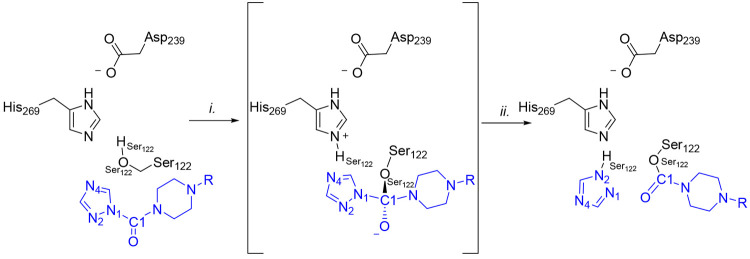
Mechanism of MGL carbamoylation by **4**: (i) TI formation through nucleophilic attack by Ser122 to the carbonyl carbon (C1) of **4**; (ii) TI decomposition prompted by triazole LG expulsion.

The Michaelis complex of **4** with MGL was thus employed to investigate the mechanism of carbamoylation at DFTB3/AMBER level.^37^ We hypothesized a reaction path in which the nucleophilic serine attacks the carbonyl carbon of the triazole urea group generating a TI, which in turn evolves expelling the triazole LG with formation of a carbamoylated serine (Figure 8).

Following the same protocol used for **1**, the MGL–**4** Michaelis complex was further equilibrated by MD at QM/MM level (400 ps), applying a DFTB3/AMBER potential.^37^ In this case, atoms treated at QM level include side chains of the catalytic triad and the (4-methylpiperazine-1-yl)(1*H*-1,2,4-triazol-1-yl) methanone fragment of **4** (see Methods for details). Analysis of the QM/MM MD trajectory shows that the urea functional group of **4** does not experience a significant deplanarization during the simulation. Geometric parameters, already applied to describe distortion from planarity of tertiary ureas,^50^ such as the dihedral angle δ and the improper torsion θ (see Table S2 for details) describing the rotation of the C1–N1 bond and the pyramidalization of the N1 nitrogen respectively, assume average values close to 0 degrees.

The QM/MM equilibrated structure was employed to simulate MGL carbamoylation by progressively converting the Michaelis complex into the carbamoylenzyme. The RC was defined as the difference of two distances, the first accounting for the nucleophilic attack [*d*(O_Ser122_, C1)] and the second describing the expulsion of the LG [*d*(C1, N1)]. A first path for MGL carbamoylation was obtained forcing the progression of the system along the RC by SMD (Figure S6). Analysis of the reaction trajectory shows that the RC can capture all relevant events required to generate a carbamoylated product: (i) activation of the nucleophile Ser122 by His269, (ii) nucleophilic attack to the carbonyl carbon with generation of a TI, and (iii) expulsion of a triazole anion with formation of the carbamoylated Ser122 followed by triazole protonation. We thus extracted a set of geometries along the path connecting reactants and products, and we used them as starting points for US simulations.^36^ The PMF was thus reconstructed applying the WHAM approach.^44^

**Figure 9:**
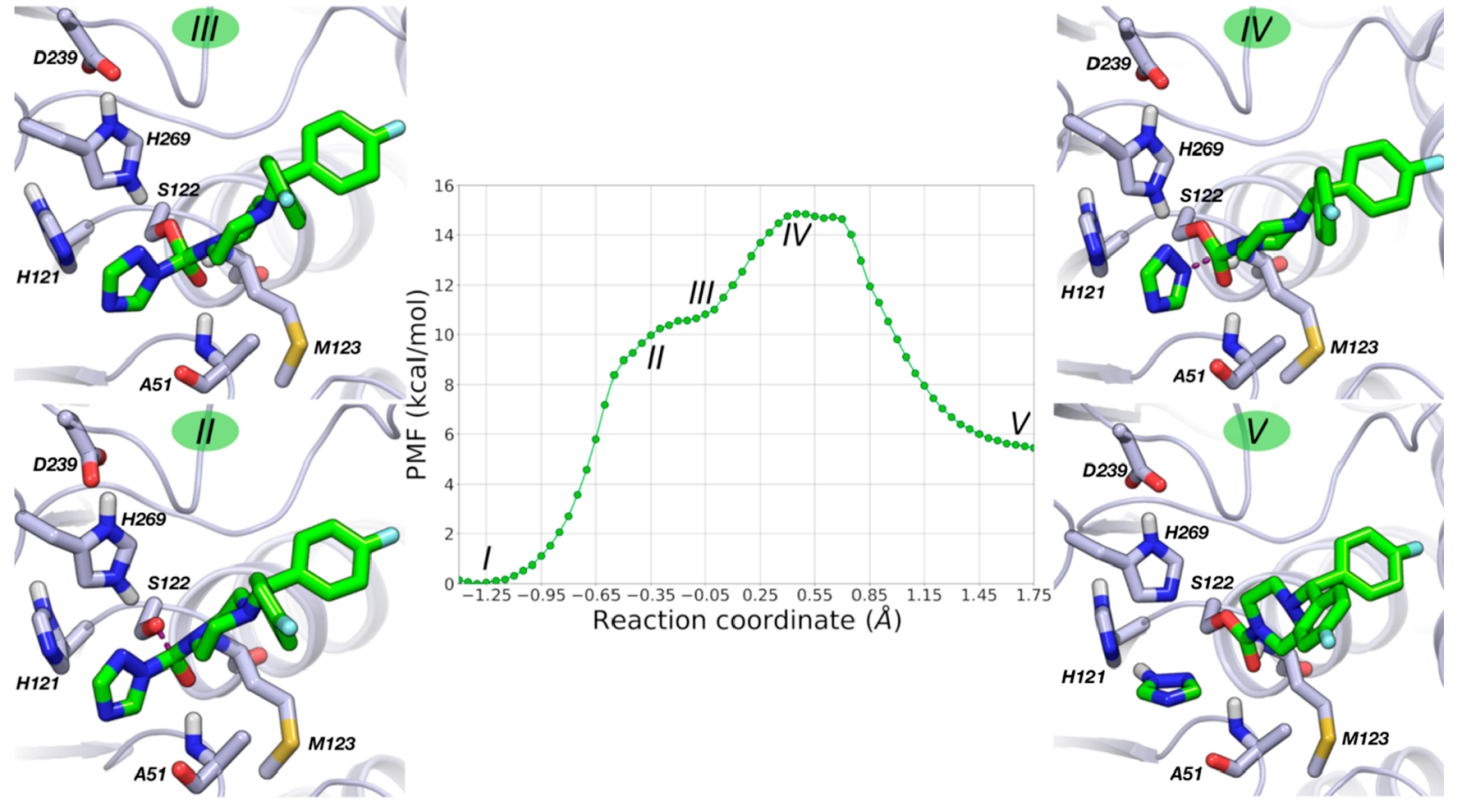
PMF for MGL carbamoylation calculated at the DFTB3/AMBER level (500 ps of simulation for each US window). Free-energy values are given in kcal·mol^–1^, and the RC is given in Å. Relevant configurations along the reaction pathway are represented: (II) nucleophilic attack by Ser122 (RC = −0.50 Å); (III) TI (RC = −0.05 Å); (IV) TS (RC = 0.475 Å); (V) carbamoylenzyme with the expelled triazole ring (RC = 1.75 Å). **4** is represented with light green carbon atoms and MGL with gray carbon atoms.

The PMF of MGL carbamoylation (after 500 ps of US simulation per window) is reported in Figure 9. Convergence of the PMF is achieved after 400 ps for each US window (Figure S7), and the uncertainty in the estimation of the free energy is nearly 0.2 kcal·mol^–1^.

MGL carbamoylation by **4** is a tightly concerted mechanism. Starting from the Michaelis complex (I on the PMF, Figure 9), the reaction initiates with the deprotonation of the catalytic Ser122 by His269, which leads to an alcoholate/imidazolium pair. This event triggers the nucleophilic attack (configuration II, Figure 9) and leads to the formation of a TI (configuration III, Figure 9). Analysis of the geometries connecting configurations I and III indicates (Table 3) that as the O_Ser122_–C1 bond becomes shorter, the C1–O (carbonyl) bond lengthens reaching a value of 1.34 ± 0.03 Å, typical of an oxyanion species. Analysis of the Mulliken charges of QM atoms confirms a significant variation in the electronic structure of the inhibitor (Table 4), with charges for C1 and O changing from the values of 0.64 ± 0.01 (C1) and −0.80 ± 0.03 (O) in the Michaelis complex (I) to 0.86 ± 0.02 (C1) and −1.08 ± 0.04 (O) in the TI (III). The change in the electronic structure of the carbonyl carbon C1 induces a reorientation of the triazole ring that becomes able to form a H-bond with the imidazolium ring of His269 through its N2 atom. While the TI (III) is well stabilized by a network of H-bonds, this configuration is not a minimum. The reaction thus proceeds with the collapse of the TI driven by the expulsion of an anionic triazole. This event represents the main TS of the reaction (RC = 0.475 Å, configuration IV, Figure 9), and it requires an energy of approximately 15 kcal·mol^–1^ above the level of the reactants (I). Analysis of TS structures indicates that in this configuration the breakage of the C1–N1 bond is occurring (1.92 ± 0.04 Å), with the incoming negative charge delocalized on the nitrogen atoms of the triazole ring (Table 4). The TS is characterized by an electrostatic interaction between the N2 atom of the triazole ring and His269 (N2–H_Ser122_ distance 2.66 ± 0.37 Å). Once the breakage of the C1–N1 bond is completed, protonation of the triazole N2 atom by His269 can occur giving the carbamoylenzyme (configuration V, Figure 9). The generation of configuration V is accompanied by a progressive change in the orientation of the 1-(bis(4-fluorophenyl)methyl)piperazine fragment, which, in the carbamoylenzyme, assumes the same orientation observed in the X-ray structure of **4** covalently bound to MGL (Figure S8). Additionally, the free triazole ring forms a H-bond with His121, similarly to that observed for glycerol in the case of acylation by 2-AG substrate (**1**).

**Table 3:** Distances^a^ between Key Atoms Involved in MGL Carbamoylation by **4**

	O_Ser122_–C1	C1–N1	C1–O	O_Ser122_–H_Ser122_	N_His269_–H_Ser122_	O_Asp239_–H_His269_	N2–H_Ser122_
Michaelis complex (I)	2.69 ± 0.06	1.44 ± 0.03	1.28 ± 0.02	0.99 ± 0.03	1.95 ± 0.20	1.81 ± 0.10	4.07 ± 0.53
II	2.09 ± 0.09	1.49 ± 0.04	1.29 ± 0.02	1.13 ± 0.24	1.65 ± 0.32	1.79 ± 0.11	2.88 ± 0.36
TI (III)	1.57 ± 0.05	1.51 ± 0.03	1.34 ± 0.03	1.97 ± 0.14	1.04 ± 0.03	1.66 ± 0.10	2.52 ± 0.25
TS (IV)	1.44 ± 0.04	1.92 ± 0.04	1.30 ± 0.02	2.25 ± 0.20	1.02 ± 0.03	1.69 ± 0.09	2.66 ± 0.37
carbamoylenzyme (V)	1.36 ± 0.03	3.11 ± 0.06	1.28 ± 0.02	4.03 ± 0.29	3.12 ± 0.42	1.86 ± 0.10	1.03 ± 0.03

a Reported as average ± SD in Å.

**Table 4:** Charges^a^ for Key Atoms Involved in MGL Carbamoylation by **4**

	C1	O	N1	N2	N4	O_Ser122_	N_His269_
Michaelis complex (I)	0.64 ± 0.01	–0.80 ± 0.03	0.12 ± 0.03	–0.34 ± 0.02	–0.41 ± 0.04	–0.61 ± 0.02	–0.42 ± 0.02
II	0.71 ± 0.03	–0.85 ± 0.04	0.11 ± 0.03	–0.33 ± 0.03	–0.45 ± 0.04	–0.61 ± 0.08	–0.33 ± 0.14
TI (III)	0.86 ± 0.02	–1.08 ± 0.04	0.12 ± 0.03	–0.37 ± 0.03	–0.50 ± 0.04	–0.57 ± 0.03	–0.03 ± 0.02
TS (IV)	0.87 ± 0.02	–0.97 ± 0.03	–0.13 ± 0.04	–0.38 ± 0.02	–0.57 ± 0.03	–0.49 ± 0.02	–0.02 ± 0.02
carbamoylenzyme (V)	0.80 ± 0.01	–0.84 ± 0.03	–0.33 ± 0.02	0.07 ± 0.03	–0.48 ± 0.04	–0.37 ± 0.02	–0.40 ± 0.02

a Reported as average ± SD in atomic unit (au).

### Molecular Models of MGL–**5** and MGL–**6** Michaelis Complexes

The mechanism of inhibition of MGL by piperazine pyrazole ureas **5** and **6** was investigated using the same multiscale approach applied for **1** and **4**. Docking simulations provided two alternative poses (with similar score) for both **5** and **6**, corresponding to (*E*)- or (*Z*)-configuration of the urea group. Similarly to what was reported for triazole **4**, gas phase calculations indicate that the (*E*)-isomer is more stable than the (Z)-isomer by several kcal·mol^–1^ (Table S1).

**Figure 10:**
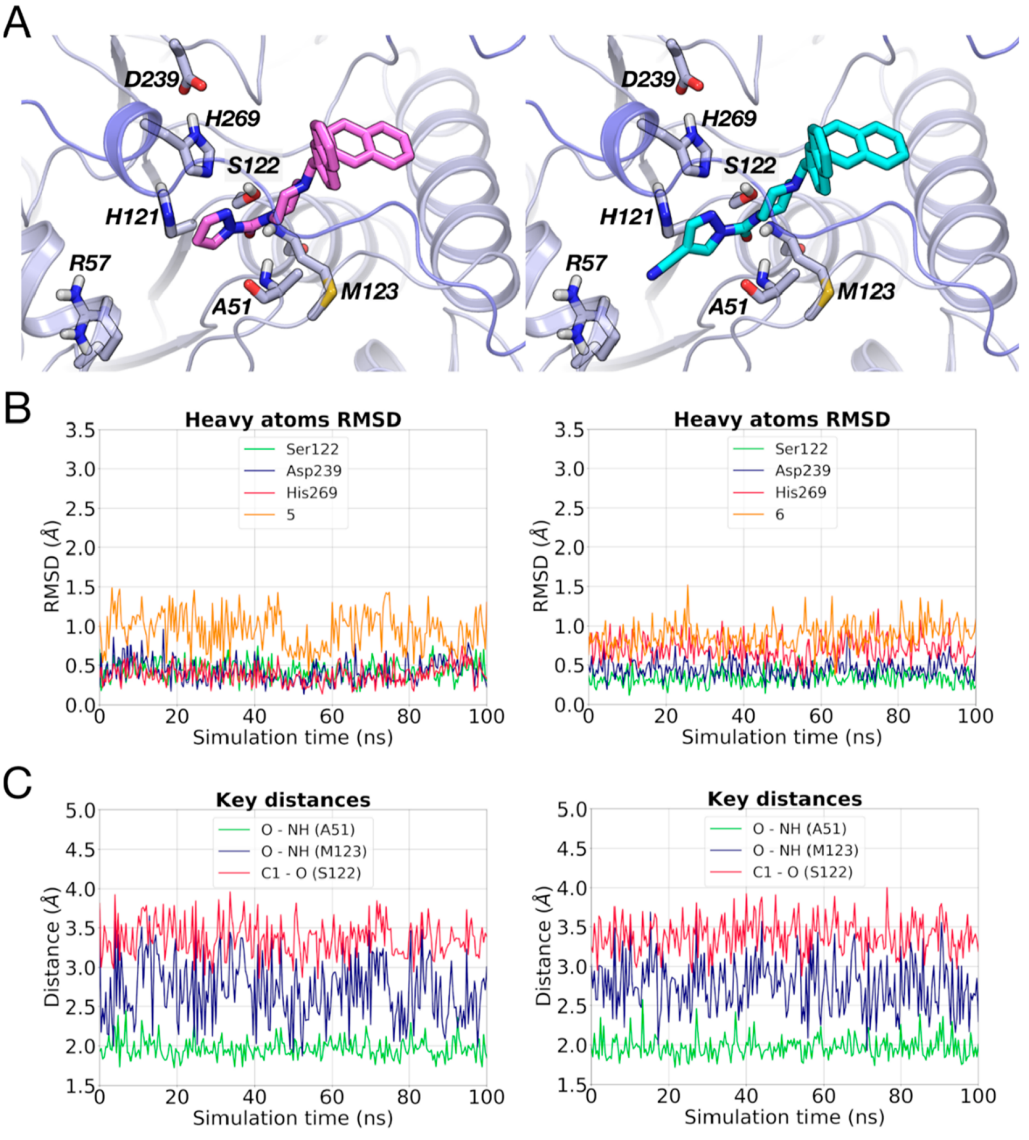
(A) Docking poses of **5** (pink carbon atoms) and **6** (cyan carbon atoms) within MGL (gray carbon atoms). (B) RMSD analysis for the heavy atoms of the catalytic residues and of **5** (left) and **6** (right) during a MD simulation. (C) Interatomic distances between the carbonyl oxygen (O) of **5** (left) or **6** (right) and the NH groups of Ala51 and Met123 and between carbonyl carbon (C1) of **5** (left) or **6** (right) and nucleophilic oxygen of Ser122 recorded during a MD simulation.

The top-ranked docking poses of **5** and **6** in the (*E*)-configuration show that these inhibitors adopt the same binding mode as **4** (Figures 10A and S9) with a similar docking score (Table S3). MD simulations (100 ns) of the Michaelis complexes show a stable arrangement of the triad (Figures 10B and S9) with pyrazoles **5** and **6** fluctuating around their docking poses (RMSD = 0.96 ± 0.24 Å for **5** and RMSD = 0.88 ± 0.17 Å for **6**) and forming stable interactions with the oxyanion hole (Figure 10C). The carbonyl carbon of the inhibitors remains at a suitable distance for the nucleophilic attack by Ser122 for the whole MD simulation (3.37 ± 0.23 Å for **5** and 3.39 ± 0.17 Å for **6**, Figure 10C). Comparable results were obtained in two other MD replicas for each system.

### Catalytic Mechanisms for MGL Carbamoylation by **5** and **6**

**Figure 11:**
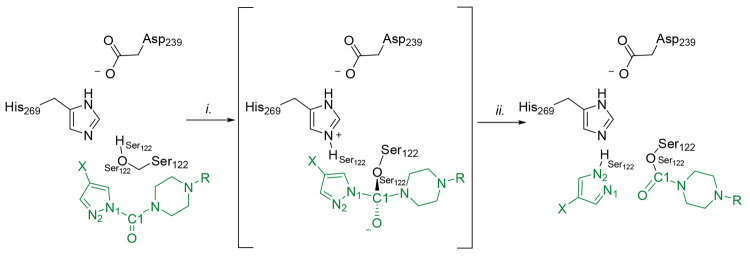
Mechanism of MGL carbamoylation by **5** and **6**: (i) TI formation through nucleophilic attack by Ser122 to the carbonyl carbon (C1) of **5** and **6**; (ii) TI decomposition prompted by pyrazole LG expulsion. X represents a hydrogen atom for **5** or a cyano group for **6**.

Following the same protocol used for **4**, the Michaelis complexes involving **5** and **6** were further equilibrated by MD at QM/MM level (400 ps), applying a DFTB3/AMBER potential (Figure 11). The QM region involves the side chains of the catalytic triad and a (4-methylpiperazine-1-yl)(1*H*-pyrazol-1-yl) methanone fragment in the case of inhibitor **5** and a (4-methylpiperazine-1-yl)(4-cyano-1*H*-pyrazol-1-yl) methanone fragment in the case of inhibitor **6**.

Analysis of the QM/MM MD trajectories shows that the urea functional group of **5** and **6** does not experience a significant distortion from planarity (Table S2). Different from what has been reported for other tertiary ureas acting on FAAH,^50^ conformational fluctuations of the enzyme active site do not seem to promote catalysis in the case of azoles **4**–**6**.

**Figure 12:**
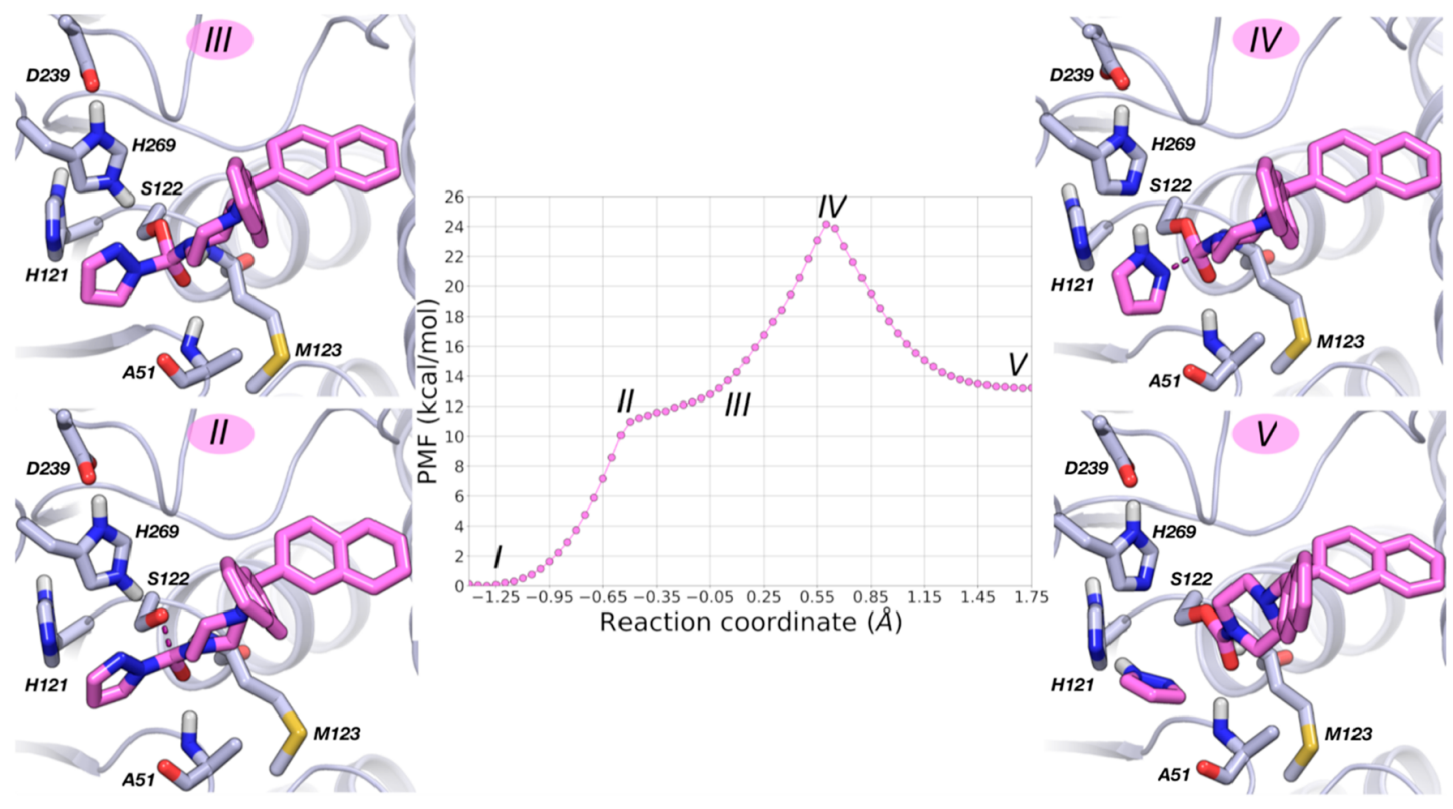
PMF for MGL carbamoylation by **5** calculated at the DFTB3/AMBER level (500 ps of simulation for each US window). Free-energy values are given in kcal·mol^–1^, and the RC is given in Å. Relevant configurations along the reaction pathway are represented: (II) nucleophilic attack by Ser122 (RC = −0.50 Å); (III) TI (RC = −0.05 Å); (IV) TS (RC = 0.625 Å); (V) carbamoylenzyme with the expelled pyrazole ring (RC = 1.75 Å). **5** is represented with pink carbon atoms and MGL with gray carbon atoms.

**Figure 13:**
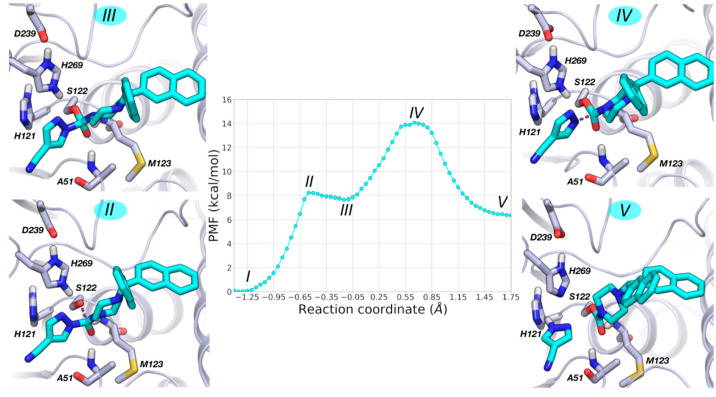
PMF for MGL carbamoylation by **6** calculated at the DFTB3/AMBER level (500 ps of simulation for each US window). Free-energy values are given in kcal·mol^–1^, and the RC is given in Å. Relevant configurations along the reaction pathway are represented: (II) nucleophilic attack by Ser122 (RC = −0.50); (III) TI (RC = −0.05); (IV) TS (RC = 0.625 Å); (V) carbamoylenzyme with the expelled 4-cyanopyrazole ring (RC = 1.75 Å). **6** is represented with cyan carbon atoms and MGL with gray carbon atoms.

We reconstructed the PMF for MGL carbamoylation by progressively converting the Michaelis complex into the carbamoylenzyme, using the same protocol employed for triazole **4**. Starting from the geometries collected by performing SMD simulations (Figure S10), the PMFs of MGL carbamoylation by **5** and **6** (after 500 ps of US simulation per window) were computed, and they are reported in Figures 12 and 13, respectively. Convergence of the computed PMFs is achieved after 400 ps for each US window with an estimated uncertainty of 0.3 kcal·mol^–1^ for both compounds (Figures S11 and S12).

Analysis of the PMFs indicates that MGL carbamoylation is a concerted process for both compounds. Starting from the Michaelis complex (I on the PMF, Figure 12), the reaction starts with the deprotonation of the Ser122 by His269. This event promotes the nucleophilic attack by Ser122 leading to the formation of a TI (configuration III, Figures 12 and 13 for **5** and **6**, respectively). Analysis of the geometries connecting I and III indicates that as the O_Ser122_–C1 bond becomes shorter, the C1–O bond lengthens reaching a value of 1.34 ± 0.03 Å at the TI (Tables 5 and 6). Mulliken charge analysis confirms a change in the electronic structure of the QM atoms with a variation in the magnitude of the charges for C1 and O nearly identical for both pyrazole inhibitors (Tables 7 and 8). The reaction proceeds with the collapse of the TI due to the expulsion of the pyrazole ring, which represents the main TS of the reaction (RC = 0.625 Å, configuration IV, Figures 12 and 13 for **5** and **6**, respectively). The free energy required to reach the TS for MGL carbamoylation is 24 kcal·mol^–1^ for **5** and 14 kcal·mol^–1^ for **6**.

**Table 5:** Distances^a^ between Key Atoms Involved in MGL Carbamoylation by **5**

	O_Ser122_–C1	C1–N1	C1–O	O_Ser122_–H_Ser122_	N_His269_–H_Ser122_	O_Asp239_–H_His269_	N2–H_Ser122_
Michaelis complex (I)	2.67 ± 0.06	1.42 ± 0.03	1.28 ± 0.02	0.99 ± 0.03	1.95 ± 0.18	1.82 ± 0.11	3.60 ± 0.57
II	2.12 ± 0.06	1.48 ± 0.03	1.28 ± 0.02	1.02 ± 0.08	1.83 ± 0.18	1.81 ± 0.12	2.70 ± 0.23
TI (III)	1.59 ± 0.05	1.50 ± 0.04	1.34 ± 0.03	1.94 ± 0.19	1.04 ± 0.03	1.66 ± 0.13	2.39 ± 0.21
TS (IV)	1.44 ± 0.04	2.13 ± 0.08	1.28 ± 0.02	2.89 ± 0.25	2.11 ± 0.25	1.91 ± 0.16	1.03 ± 0.03
carbamoylenzyme (V)	1.35 ± 0.03	3.10 ± 0.06	1.28 ± 0.02	3.83 ± 0.40	3.05 ± 0.51	1.87 ± 0.14	1.03 ± 0.03

a Reported as average ± SD in Å.

**Table 6:** Distances^a^ between Key Atoms Involved in MGL Carbamoylation by **6**

	O_Ser122_–C1	C1–N1	C1–O	O_Ser122_–H_Ser122_	N_His269_–H_Ser122_	O_Asp239_–H_His269_	N2–H_Ser122_
Michaelis complex (I)	2.68 ± 0.06	1.42 ± 0.03	1.28 ± 0.02	0.98 ± 0.02	2.04 ± 0.24	1.83 ± 0.11	3.08 ± 0.24
II	2.00 ± 0.09	1.47 ± 0.04	1.29 ± 0.02	1.50 ± 0.35	1.31 ± 0.38	1.74 ± 0.11	2.66 ± 0.21
TI (III)	1.58 ± 0.05	1.52 ± 0.04	1.34 ± 0.03	1.95 ± 0.16	1.04 ± 0.03	1.69 ± 0.10	2.59 ± 0.22
TS (IV)	1.42 ± 0.04	2.06 ± 0.05	1.29 ± 0.03	2.39 ± 0.25	1.02 ± 0.03	1.71 ± 0.12	2.61 ± 0.31
carbamoylenzyme (V)	1.36 ± 0.03	3.12 ± 0.06	1.27 ± 0.02	3.23 ± 0.44	2.21 ± 0.34	1.94 ± 0.13	1.03 ± 0.0

a Reported as average ± SD in Å.

**Table 7:** Charges^a^ for Key Atoms Involved in MGL Carbamoylation by **5**

	C1	O	N1	N2	O_Ser122_	N_His269_
Michaelis complex (I)	0.65 ± 0.01	–0.80 ± 0.03	0.15 ± 0.03	–0.32 ± 0.02	–0.61 ± 0.02	–0.40 ± 0.02
II	0.71 ± 0.02	–0.85 ± 0.03	0.13 ± 0.02	–0.30 ± 0.02	–0.56 ± 0.03	–0.40 ± 0.04
TI (III)	0.87 ± 0.02	–1.08 ± 0.03	0.14 ± 0.03	–0.35 ± 0.02	–0.58 ± 0.02	–0.03 ± 0.02
TS (IV)	0.84 ± 0.02	–0.88 ± 0.03	–0.23 ± 0.03	0.08 ± 0.03	–0.44 ± 0.02	–0.42 ± 0.02
carbamoylenzyme (V)	0.80 ± 0.01	–0.85 ± 0.02	–0.30 ± 0.02	0.10 ± 0.02	–0.37 ± 0.03	–0.40 ± 0.02

a Reported as average ± SD in atomic unit (au).

**Table 8:** Charges^a^ for Key Atoms Involved in MGL Carbamoylation by **6**

	C1	O	N1	N2	N4^b^	O_Ser122_	N_His269_
Michaelis complex (I)	0.65 ± 0.01	–0.79 ± 0.02	0.15 ± 0.02	–0.28 ± 0.02	–0.38 ± 0.03	–0.61 ± 0.02	–0.41 ± 0.02
II	0.75 ± 0.03	–0.89 ± 0.05	0.16 ± 0.02	–0.29 ± 0.02	–0.40 ± 0.03	–0.70 ± 0.10	–0.17 ± 0.16
TI (III)	0.87 ± 0.02	–1.07 ± 0.04	0.14 ± 0.02	–0.32 ± 0.02	–0.42 ± 0.03	–0.58 ± 0.03	–0.03 ± 0.02
TS (IV)	0.86 ± 0.02	–0.93 ± 0.04	–0.15 ± 0.05	–0.34 ± 0.03	–0.46 ± 0.03	–0.46 ± 0.02	–0.02 ± 0.02
carbamoylenzyme (V)	0.80 ± 0.01	–0.84 ± 0.03	–0.28 ± 0.02	0.09 ± 0.02	–0.40 ± 0.03	–0.38 ± 0.02	–0.43 ± 0.02

a Reported as average ± SD in atomic unit (au).

b In this case, N4 refers to the terminal nitrogen of the 4-cyano group of **6**.

Analysis of the TS geometries shows that **5** and **6** follow two different reaction paths. The TSs for carbamoylation by the two pyrazoles are characterized by the breakage of the C1–N1 bond, which reached a value of 2.13 ± 0.08 Å for **5** and of 2.06 ± 0.05 Å for **6**. In the case of **5**, this event is assisted by the protonation of the LG at the N2 position with N2–H_Ser122_ distance of 1.03 ± 0.03 Å. This is not the case for the 4-cyanopyrazole **6**, in which the N2–H_Ser122_ distance (2.61 ± 0.31 Å) is consistent with that of a weak H-bond. Moreover, the 4-cyano group of **6** protrudes into the glycerol binding pocket forming a polar interaction with Arg57 (Figure S13), which likely contributes to the stabilization of the TS.

Analysis of the Mulliken charges confirms that at the TS the electronic distribution of the LG of **5** is rather different from that of **6**. In the case of **5**, N1 and N2 assume a negative and a positive charge, respectively, because of the protonation of the pyrazole at N2. In the case of **6**, all the nitrogen atoms of the 4-cyanopyrazole ring possess a negative charge due to the expulsion of an anionic LG (Table 8). For both inhibitors **5** and **6**, the generation of the carbamoylated adduct (configuration V, Figures 12 and 13) is associated with a change in the orientation of the 1-(bis(naphtalen-2-yl)methyl)piperazine fragment, which, in the final product, assumes the same pose as the 1-(bis(4-fluorophenyl)methyl)piperazine portion observed in the X-ray structure of **4** covalently bound to MGL. In configuration V, the pyrazole and the 4-cyanopyrazole are both protonated and can form a H-bond with His121, similarly to what was observed for the triazole generated during carbamoylation by **4**. Configuration of the carbamoylenzyme (V) is characterized by an energy value of 13 kcal·mol^–1^ above the reactants (I) in the case of pyrazole **5** and of 6 kcal·mol^–1^ above I in the case of 4-cyanopyrazole **6**.

**Figure 14:**
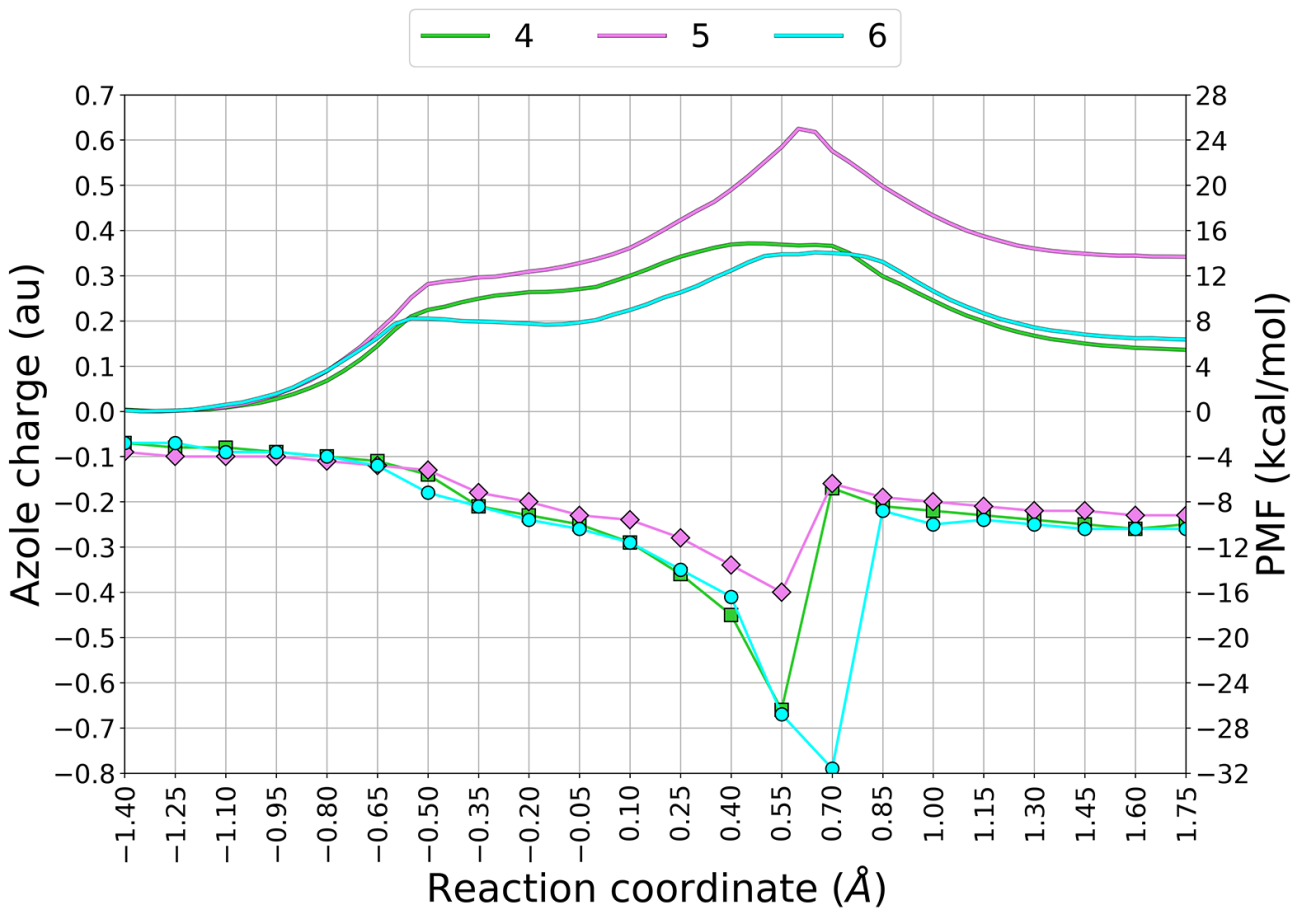
Progress along the RC of the overall Mulliken charge (calculated as average within each US window) of the azole LG of **4**–**6**. Mulliken charge values are expressed in atomic unit (au). PMF for MGL carbamoylation by **4**–**6** calculated at the DFTB3/AMBER level. Free-energy values are given in kcal·mol^–1^.

To evaluate the ability of the azole nucleus to serve as a good LG during MGL carbamoylation by compounds **4**–**6**, the overall Mulliken charge of this ring was calculated as a function of the RC and compared to the computed PMFs (Figure 14). The magnitude of the negative charge on the azole ring increases as the reaction proceeds along the RC for all three inhibitors. However, in correspondence with RC values in which the PMF for carbamoylation approaches the TS region (RC = 0.40–0.55 Å), the triazole and 4-cyanopyrazole leaving groups develop a significantly more negative charge than the pyrazole ring. The electron-withdrawing nature of the 4-aza or 4-cyano groups, being able to delocalize the incipient charge due to the breakage of the ureidic bond, seems to favor the expulsion of an anionic LG.

### Insights for the Design of New Piperazine Pyrazole Ureas

Available SAR data provided by Boger and co-workers,^20^ together with our computational findings, highlight the importance of having an electron-withdrawing substituent on the pyrazole leaving group of tertiary ureas to achieve potent MGL inhibition. In the context of covalent inhibitor design, it is conceivable that the introduction of other electron-withdrawing substituents on the pyrazole ring such as the 4-carboxamide group, the size of which appears to be tolerated by the MGL active site, might lead to the identification of compounds able to potently inhibit MGL. A pyrazole-4-carboxamide derivative has been recently described in a granted patent as a potent inhibitor of MGL (no. 204, 1-(4-(4-chlorobenzyl)piperazine-1-carbonyl)-1*H*-pyrazole-4-carboxamide, IC_50_ < 100 nM).^51^ To test our multiscale approach, calibrated using compounds **4**–**6**, we calculated the PMF of MGL carbamoylation at DFTB3/AMBER level also for this pyrazole-4-carboxamide urea (compound **7**).

**Figure 15:**
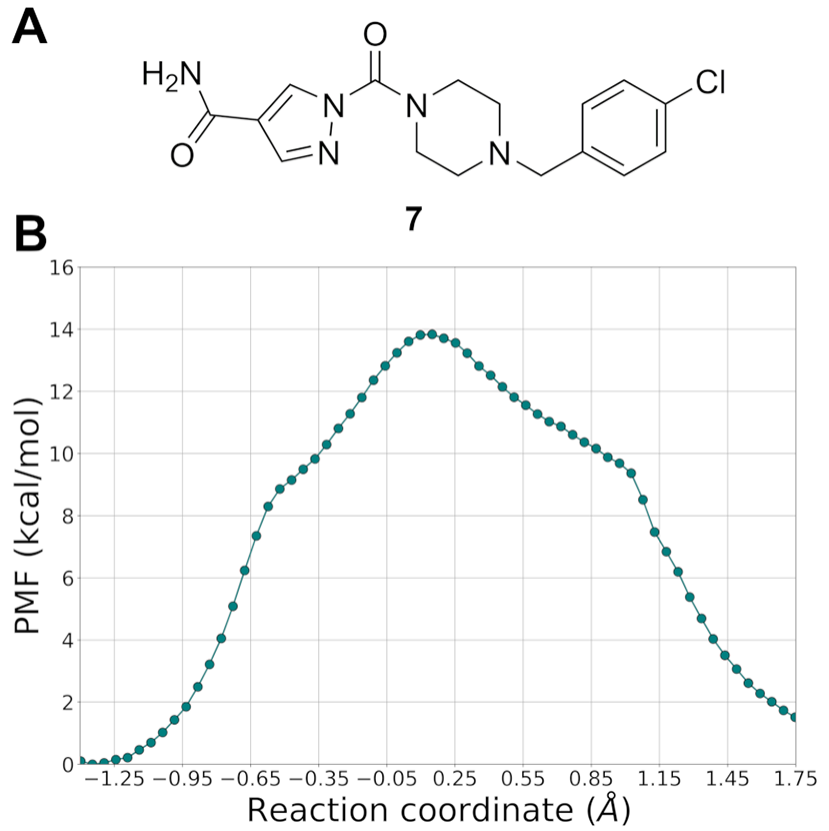
(A) Structure of compound **7**. (B) PMF for MGL carbamoylation by **7** calculated at the DFTB3/AMBER level (500 ps of simulation for each US window). Free-energy values are given in kcal·mol^–1^, and the RC is given in Å.

In brief, **7** was docked within the MGL binding site, and the top-ranked pose with the (*E*)-configuration was selected (Figure S14). The resulting MGL–**7** Michaelis complex was solvated and equilibrated by MD simulations at MM and QM/MM levels. We thus reconstructed the PMF for MGL carbamoylation by progressively converting the Michaelis complex into the carbamoylenzyme using the same protocol employed for **4**–**6**, based on SMD and US simulations at DFTB3/AMBER level and WHAM analysis. The resulting PMF (Figure 15) is characterized by a free energy barrier of nearly 14 kcal·mol^–1^, a value significantly lower than that computed for the weak MGL inhibitor **5** and in line with the barriers calculated for the potent azole inhibitors **4** and **6**.

## Discussion

MGL is a member of the serine hydrolase superfamily, endowed with a classic Ser–His–Asp triad, responsible for the deactivating cleavage of the endocannabinoid 2-AG (**1**).^1^ Compelling data indicate that **1** sustains neuroprotection and recovery from neuronal insults.^4^ These findings have promoted MGL as a promising drug target, with carbamate-based inhibitors being currently evaluated in clinical phase.^19^ From a biochemical standpoint, MGL has been largely investigated over the years, often with a focus on allosteric regulation and oxidative stress.^6,12^ Only recently, systematic investigations on the role of active site residues of MGL during the catalytic cleavage of **1** have appeared in the literature.^7^ Despite these recent advances, an atomistic understanding of the catalytic mechanism employed by MGL is still lacking.

In the present work, we have employed a QM/MM approach based on US simulations^36^ to reconstruct the free energy surfaces of relevant reactions catalyzed by MGL, namely, acylation by 2-AG (**1**) and carbamoylation by the triazole urea SAR629 (**4**) and pyrazole ureas **5** and **6**. Our investigations have a twofold scope: (i) assessing the ability of our QM/MM protocol to provide insights on MGL catalytic activity in the presence of 2-AG (**1**) and SAR629 (**4**) in agreement with mutagenesis^7^ and structural data^14^ (*vide infra*) and (ii) exploiting the same multiscale protocol in the rationalization of inhibitory potency data (i.e., IC_50_ values) of azoles **5** and **6** for which the mechanism of action is somehow unclear.^24^ In the case of covalent inhibitors, the IC_50_ value accounts for both the recognition (formation of the Michaelis complex) and the chemical step (covalent modification of an active site residue) of enzyme inhibition. The IC_50_ parameter can still give clean SAR information on the intrinsic reactivity if the recognition scaffold of the inhibitors under investigation is kept essentially constant.^13,52,53^ In light of the similarity displayed by compounds **4**–**6**, which leads to a nearly identical noncovalent binding mode, we assumed that observed differences in IC_50_ values reflect a diverse ability of the azole ureas to covalently react with MGL.

Our calculations showed that MGL acylation by **1** is a two-step process with formation of a tetrahedral intermediate (TI). The key actor of the first step is the nucleophile Ser122, which, once deprotonated by His269, attacks the carbonyl carbon of **1** giving the TI. The carboxylate of Asp239 contributes to this step by stabilizing the incoming positive charge of His269. The lack of activity displayed by D239A and D239N MGL mutants underlines the importance of a strong electrostatic interaction for an effective catalytic process.^7^ Protonation of the esterified oxygen (O2) of the glycerol by the cationic His269 is the key event of the second step. This reaction triggers the expulsion of the glycerol LG and the generation of an acylenzyme. This process is assisted by a H-bond formed by glycerol and an accessory His residue (His121) proximal to the catalytic triad. The importance of this interaction is supported by mutagenesis data with the H121A MGL variant displaying a reduced catalytic activity (10.3-fold) compared to the wild-type.^7^ It is also worth mentioning that our simulations identify a binding pose for glycerol (at the end of acylation) close to the one displayed in X-ray structures of MGL (PDB codes 6AX1(39) and 3HJU,^5^Figure S4).

The PMF of MGL acylation indicates that the reaction barriers for the two key steps, that is, formation and collapse of the TI, are similar in their magnitude with not a single event being rate-limiting. The computed barrier (10 kcal·mol^–1^) is somehow low compared to the experimental one (approximately 16–17 kcal·mol^–1^), deduced from the application of the TS theory^54^ from available *k*_cat_ values.^7,55^ This can be attributed to the DFTB3/3OB level of theory here applied, which, while able to capture fundamental aspects of catalysis including structure–reactivity relationships for substrate^56^ and inhibitors,^57,58^ underestimates reaction barriers.^59−61^ Our mechanistic description of acylation by 2-AG is consistent with data on MGL mutants^7^ and produces geometries consistent with available X-ray structures of MGL.

QM/MM simulations show that carbamoylation by triazole urea **4** occurs as a single step process, in which the collapse of the TI emerges as the difficult step of carbamoylation. The simulated mechanism appears structurally reasonable, with the geometry of the carbamoylenzyme superimposable to that observed in the X-ray structure of **4** covalently bound to MGL (Figure S8). The PMF of MGL carbamoylation by **4** and the analysis of the geometries collected at the TS indicate that the rate limiting step of this reaction is dominated by the breakage of the bond connecting the N1 atom of the triazole to the carbonyl carbon of the urea (C1–N1 bond). The computed barrier for this process is 15 kcal·mol^–1^, consistent with a fast inhibition of MGL.

We finally exploited our QM/MM protocol to elucidate the mechanism of action of two MGL inhibitors having a common 1-(bis(naphthalen-2-yl)methyl)piperazine scaffold but being equipped with two differently substituted pyrazoles, 4-H in the case of **5** and 4-cyano in the case of **6**. These two compounds possess different potency on MGL, with **5** and **6** being active in the micromolar and nanomolar range, respectively. The observed difference in their inhibitory potency can be ascribed to the diverse propensity of **5** and **6** to carbamoylate Ser122. QM/MM simulations showed that also for pyrazole ureas carbamoylation is a concerted process, where the expulsion of the pyrazole LG represents the rate-limiting step of the reaction. Analysis of the PMF shows that carbamoylation by **5** requires overcoming a high free energy barrier (24 kcal·mol^–1^), ∼10 kcal·mol^–1^ over the barrier calculated for the reference triazole **4**. In the case of carbamoylation by the 4-cyanopyrazole **6**, the activation energy is 14 kcal·mol^–1^, significantly lower than that of pyrazole **5** and in line with that of triazole **4**. Analysis of the reaction geometries indicates that the two pyrazole ureas follow two different reactions paths. In the case of **5**, the breakage of the C1–N1 bond is assisted by the protonation of the pyrazole ring at the N2 atom by His269. This event allows the expulsion of a neutral pyrazole generating the final carbamoylenzyme. In the case of **6**, the breakage of the C1–N1 bond does not require protonation at N2, thus the reaction occurs with the expulsion of an anionic 4-cyanopyrazole LG. The presence of a substituent with electron-withdrawing properties at the 4-position drives the reaction versus a more accessible reaction path with a TS in which the incoming negative charge on the LG is delocalized among all the heteroatoms. Simulations also point to a specific interaction undertaken by the 4-cyano group with Arg57, which likely contributes to a further stabilization of the main TS of carbamoylation. However, we cannot exclude that this polar interaction may also have a positive effect on the recognition step. Arg57 is an accessible polar spot in MGL that can be exploited for the design of further inhibitors, as revealed by the recent release of X-ray structures of MGL in complex with inhibitors featuring a benzo[*b*][1,4]oxazine moiety.^62^

Overall, our simulations indicate that while N2 protonation of the pyrazole nitrogen is an event that favors carbamoylation, a better outcome in terms of chemical reactivity is obtained through the introduction of an electron-withdrawing substituent on the pyrazole nucleus, which allows the expulsion of an anionic LG. Modulation of the electronic state of the pyrazole nucleus emerges as an effective strategy to tune the inhibitory activity of MGL inhibitors. Computational results obtained with compound **7**, bearing a 4-carboxamide substituent on the pyrazole leaving group, further support this insight.

## Conclusions

In this work, we applied a QM/MM approach coupled to enhanced sampling methods to elucidate the catalytic mechanism of MGL in the presence of 2-AG and both triazole and pyrazole urea inhibitors. The results of our simulations support the hypothesis that the expulsion of the leaving group is an important event of both acylation and carbamoylation of Ser122. The inhibitory activity of pyrazole urea compounds can be modulated by substituting the azole ring with electron-withdrawing groups able to delocalize the incipient negative charge during the breakage of the C–N bond. The ability of our multiscale approach to distinguish nanomolar from micromolar MGL inhibitors will be exploited in the design of agents targeting Ser122 with a fine-tuned reactivity.

## Methods

### Preparation of the Protein–Ligand Models and MM Simulations

The models of MGL in complex with **1** and **4**–**7** were built starting from chain B of the X-ray structure of hMGL covalently bound to **4** (PDB code 3JWE).^14^ The experimental potencies of the selected inhibitors were obtained on mouse MGL, which has 85% of identity with hMGL. In addition, 99% of the residues situated 6 Å from the Ser122–**4** adduct are identical. This high conservation of the protein sequence among the two forms of the enzyme allows us to model the reaction starting from the crystal structure of the human isoform of MGL.

The two molecules of **4** cocrystallized in chain B were removed, the catalytic serine (Ser122) was restored, and the protein was refined using the Protein Preparation Wizard tool^63^ available in Maestro 11.6.^64^ Hydrogen atoms were added, and the orientation of hydroxyl groups and conformations of asparagine and glutamine side chains were adjusted in order to maximize the number of hydrogen bonds. Acid and basic residues were modeled in their negatively and positively charged forms, respectively. The histidine residues were maintained in their neutral form, and for each residue, the tautomer was selected to maximize the polar interactions. The obtained structure was submitted to a restrained minimization using the OPLS3e force field,^65^ in which only the hydrogen atoms were free to move. A second minimization was performed, in which also heavy atoms were free to move up to an RMSD value of 0.3 Å.

Michaelis-like complexes of MGL and **1** and **4**–**7** were generated by docking using Glide 7.9.^66,67^ The structures of **1** and **4**–**7** were built in Maestro and prepared with the LigPrep tool.^68^ The docking grid was centered on the center of mass of residues Ala51, Ser122, Met123, and His269, and the inner and outer box dimensions were set, respectively, to 16 and 36 Å for 1 and 13 and 33 Å for **4**–**7**. H-bond constraints between the oxyanion hole nitrogen of Ala51 and Met123 and the carbonylic oxygen atom of **1** and **4**–**7** were added. Docking studies were performed using the standard precision (SP) mode and were forced to satisfy at least one of the two previously defined hydrogen bonds. All remaining parameters were applied as default. Gscore values were used to select the best docking poses. MacroModel tool 12.0^69^ was used to minimize the resulting Michaelis complexes by applying OPLS3e force field^65^ keeping the α carbon atoms of the protein fixed. Distance constraints were also used to maintain the H-bond interactions with the oxyanion hole. The resulting structures were imported in t-leap for parametrization with AMBER. In detail, the AMBERff15ipq^70^ force field and general AMBER force field (GAFF)^41^ were applied to model the protein and the ligands, respectively. Each protein–ligand complex was solvated into a simulation box of 83 Å × 70 Å × 69 Å by adding TIP3P water molecules (9315 for MGL–**1**, 9312 for MGL–**4**, 9311 for MGL–**5** and MGL–**6**, and 9324 for MGL–**7**)^71^ and neutralized by 3 Na^+^ ions. The systems were further minimized with the AMBER force field and equilibrated for 2.5 ns under NVT and for 13 ns under NPT conditions, increasing the temperature up to 298 K and gradually reducing constraints on both the ligand and the protein. Hydrogen atoms were handled with the SHAKE algorithm, and a cutoff of 10 Å was selected to treat the electrostatic and van der Waals interactions. Long-range electrostatic interactions were treated using the particle mesh Ewald (PME) method. The production phase was carried out for 100 ns under NVT conditions. Harmonic restraints of 5 kcal·mol^–1^·Å^–2^ were applied on the α carbon atoms of residues situated 5 Å from the active site region to maintain the lid domain in its open conformation, which is believed to represent the catalytically competent form of MGL (see SI for a detailed list of restrained atoms). For each Michaelis complex three independent replicas of 100 ns were performed by using the *pmemd* module of the AMBER16 software.^72^

### Application of the QM/MM Potential

For each MGL–ligand complex, an equilibrated snapshot taken from the last 20 ns of the MD simulation was used as starting point for hybrid QM/MM calculations. In the case of the MGL–**1** complex, the side chains of Ser122, Asp239, and His269 and the 1-hydroxy-1,3-dihydroxypropan-2-yl propionate fragment of **1** represented the *reactive region* of the system (Figure S17) and were treated with the self-consistent charge density functional tight binding type 3 (DFTB3)^47^ level of QM theory using 3OB parameters. In the case of MGL complexed with azole inhibitors **4**–**7**, the reactive region always described at DFTB3 level comprised Ser122, Asp239, and His269 side chains and alternatively included (4-methylpiperazine-1-yl)(1*H*-1,2,4-triazol-1-yl) methanone for compound **4**, (4-methylpiperazine-1-yl)(1*H*-pyrazol-1-yl) methanone for **5**, (4-methylpiperazine-1-yl)(4-cyano-1*H*-pyrazol-1-yl) methanone for **6**, and (4-methylpiperazine-1-yl)(4-carboxamide-1*H*-pyrazol-1-yl) methanone for **7** (Figure S17).

The SCC-DFTB method,^73^ of which DFTB3/3OB represents the most recent implementation, can be used to model chemical reactions occurring in enzymes as it provides results in qualitative agreement with experimental data or calculations at high level of theory at an acceptable computational cost.^74−76^ The DFTB-based approach suffers from errors in estimating weak interaction energetics, including hydrogen bonding and dispersion forces.^77^ On the other hand, the DFTB approach has been shown to give reliable results in the case of acylation and deacylation reactions on several other serine hydrolases.^31,74^

All the other atoms constituting the nonreactive region were modeled with the AMBER15ffipq force field.^70^ The Hamiltonian of the system was described by the additive QM/MM scheme:
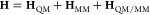
1where **H**_QM_ is the Hamiltonian of the *reactive region*, **H**_MM_ is the Hamiltonian of the *nonreactive region*, and **H**_QM/MM_ is the Hamiltonian that collects the interaction terms between the two regions, including the electrostatic interactions. The latter are computed by applying the electrostatic-embedding approach that allows inclusion of the classical point charges of the MM atoms in the QM Hamiltonian. An exhaustive illustration of the QM/MM approach employed here can be found in ref (37).

Before running enhanced sampling simulations, each system was submitted to a geometry minimization at DFTB3/AMBER level by applying the steepest descent (SP) method for 500 steps, followed by the conjugate gradient (CG) algorithm to an energy gradient of 0.005 kcal·mol^–1^·Å^–2^. Then, the optimized systems were submitted to QM/MM MD simulations of 200 ps in NVT conditions (298 K) in which a time step of 0.2 fs was used to integrate the equation of motion. In these simulations, full electrostatic and van der Waals interactions were computed within a cutoff of 10 Å, and long-range electrostatic interactions were treated using PME method. The SHAKE option was turned off during all QM/MM simulations. To maintain the protein structure close to the X-ray coordinates, harmonic restraints of 5 kcal·mol^–1^·Å^–2^ were applied on α carbon atoms of residues situated 5 Å from the active site region (see SI for a detailed list of restrained atoms).

### QM/MM Modeling of MGL Acylation by **1**

A snapshot extracted from a 200 ps long QM/MM MD simulation was used to perform a SMD simulation^43^ modeling the acylation reaction of MGL by **1**. The simulation was carried out using the sander module of AMBER16 coupled with PLUMED 2.4.1.^78^ The acylation process was described by a RC derived from the linear combination of two distances that take into account the nucleophilic attack by Ser122 on the ester carbon of **1** and the expulsion of the glycerol LG: RC = −*d*(O_Ser122_, C1) + *d*(C1, O2). The value of the RC ranged from −1.40 Å to 1.75 Å, and a harmonic restraint of 100 kcal·mol^–1^·Å^–2^ was applied to drive the system from the Michaelis complex (reactants) to the acylenzyme (products) with a constant velocity of 0.01 Å·ps^–1^. Twenty-two different geometries, chosen to represent the entire reaction path and extracted at an interval of the RC values having width of 0.15 Å, were taken from the SMD trajectory and used as starting geometries for QM/MM US simulations.^36^ Each window consisted of 13 ps of equilibration, followed by 500 ps of production dynamics, restraining the RC value by applying a harmonic restraint of 100 kcal·mol^–1^·Å^–2^. The WHAM approach^44^ was used to reconstruct the PMF^38^ by combining the results of each simulation and binning at an interval of 0.05 of the RC values. Once the PMF convergence was achieved, the uncertainty of the reported PMF was calculated by averaging the free energy values of each window retrieved at 400, 450, and 500 ps of US simulations. To characterize the geometries corresponding to the highest point of the PMF for MGL acylation by **1** and to compute the charge analysis for this state of the reaction path, we performed 500 ps long MD simulation at DFTB3/AMBER level restraining the reaction coordinate to the value of −0.125 Å, which is between the two RC values limiting the saddle point (RC = −0.15 Å and RC = −0.10 Å).

### QM/MM Modeling of MGL Carbamoylation by Azole Ureas **4**–**7**

For each system, a snapshot extracted from a 400 ps long QM/MM MD simulation was used to perform a SMD simulation^43^ describing the carbamoylation reaction of MGL. The simulations were carried out using the sander module of AMBER16 coupled with PLUMED 2.4.1.^78^ The carbamoylation process was described by a RC derived from the linear combination of two distances that take into account the nucleophilic attack by Ser122 on the urea carbon of **4**–**7** and the expulsion of the azole LG: RC = −*d*(O_Ser122_, C1) + *d*(C1, N1). The value of the RC ranged from −1.40 Å to 1.75 Å, and a harmonic restraint of 100 kcal·mol^–1^·Å^–2^ was applied to drive the system from the Michaelis complex (reactants) to the carbamoylenzyme (products) with a constant velocity of 0.01 Å·ps^–1^. Twenty-two different geometries, chosen to represent the entire reaction path and extracted at an interval of the RC values having width of approximately 0.15 Å, were taken from the SMD trajectory and used as starting geometries for QM/MM US simulations.^36^ Each window consisted of 13 ps of equilibration, followed by 500 ps of production dynamics, restraining the RC value by applying a harmonic restraint of 100 kcal·mol^–1^·Å^–2^. The WHAM approach^44^ was used to reconstruct the PMF^38^ by combining the results of each simulation and binning at an interval of 0.05 of the RC values. Once the PMF convergence was achieved, the uncertainty of the reported PMF was calculated by averaging the free energy values of each window retrieved at 400, 450, and 500 ps of US simulation. To characterize the geometries corresponding to the highest point of the PMF for MGL carbamoylation by **4**–**6** and to compute the charge analysis for this state of the reaction path, we performed 500 ps long MD simulation at DFTB3/AMBER level restraining the reaction coordinate for MGL–**4** to the value of 0.475 Å, which is between the two RC values limiting the saddle point (RC = 0.40 Å and RC = 0.55 Å), and for MGL–**5** and MGL–**6** to the value of 0.625 Å, which is between the two RC values limiting the saddle point (RC = 0.55 Å and RC = 0.70 Å).

### Data and Software Availability

All data are available upon reasonable request to the corresponding author. Schrödinger Suite 2018 (https://www.schrodinger.com) is distributed under license. AmberTools and Amber16 (https://ambermd.org) packages are available under license. PLUMED (https://www.plumed.org/) is an open-source plugin.

## Supporting Information Available

Detailed analysis of MD simulations with AMBER and detailed information on enhanced sampling simulations (SMD and US) at DFTB3/AMBER level (PDF)
Docking results for compounds **1** and **4**–**7** (ZIP)
Input files of the equilibration and production protocols of MD, SMD, and US simulations (ZIP)
Geometries of key configurations along the reaction coordinate for acylation by **1** and carbamoylation by **4**–**7** (ZIP)

## Abbreviations Used

MGL: monoglyceride lipase
MAGL: monoacylglycerol lipase
2-AG: 2-arachidonoyl-*sn*-glycerol
QM/MM: quantum mechanics/molecular mechanics
LG: leaving group
SMD: steered molecular dynamics
US: umbrella sampling
WHAM: weighted histogram analysis method
PMF: potential of mean force.
