# Supplementary material for: Mechanistic Modeling of Monoglyceride Lipase Covalent Modification Elucidates the Role of Leaving Group Expulsion and Discriminates Inhibitors with High and Low Potency
Source: J Chem Inf Model. 2022 May 17;62(11):2771–87. doi: 10.1021/acs.jcim.2c00140 (PMC9198976; doi:10.1021/acs.jcim.2c00140)
Supplement: Supplementary file 1 — ci2c00140_si_001.pdf [file ci2c00140_si_001.pdf]

Supporting Information

Mechanistic Modeling of Monoglyceride Lipase Covalent Modification Elucidates the Role of Leaving Group Expulsion and Discriminates Inhibitors with High and Low Potency

Francesca Galvani,[1] Laura Scalvini,[1] Silvia Rivara,[1] Alessio Lodola,[1*] and Marco Mor[1,2]*

[1]Dipartimento di Scienze degli Alimenti e del Farmaco, Università degli Studi di Parma, Parco Area delle Scienze 27/A, I-43124 Parma, Italy

[2]Microbiome Research Hub, University of Parma, Parco Area delle Scienze 11/A, I-43124 Parma, Italy

Table of Contents

Molecular dynamics (MD) simulation of MGL–1 3

Steered MD simulation for MGL acylation by 1. 4

Potential of mean force for MGL acylation by 1. 5

Superposition of MGL acylated by 1 and two X-ray structures of MGL and a glycerol molecule.6

Gas-phase energy calculations on (*E*) and (*Z*)-configurations for piperazine azole urea fragments taken from compound 4–6. 7

MD simulation of MGL–4. 8

Deplanarization of azole ureas 4–6 during QM/MM MD simulations. 9

Steered MD simulation for MGL carbamoylation by 4. 10

Potential of mean force for MGL carbamoylation by 4. 11

Superposition of MGL carbamoylated by 4 and the X-ray structure of MGL–4. 12

Docking scores for compounds 4–6. 13

Molecular dynamics simulations of MGL–5 and MGL–6. 14

Steered MD simulation for MGL carbamoylation by 5 and 6. 15

Potential of mean force for MGL carbamoylation by 5. 16

Potential of mean force for MGL carbamoylation by 6. 17

Polar interaction between 6 and Arg57 during US simulations. 18

Docking model of 7 within MGL binding site. 19

Steered MD simulation for MGL carbamoylation by 7. 20

Potential of mean force for MGL carbamoylation by 7. 21

List of restrained residues. 22

Definition of the QM region for the Michaelis complexes under study. 23

Molecular dynamics (MD) simulation of MGL–1.

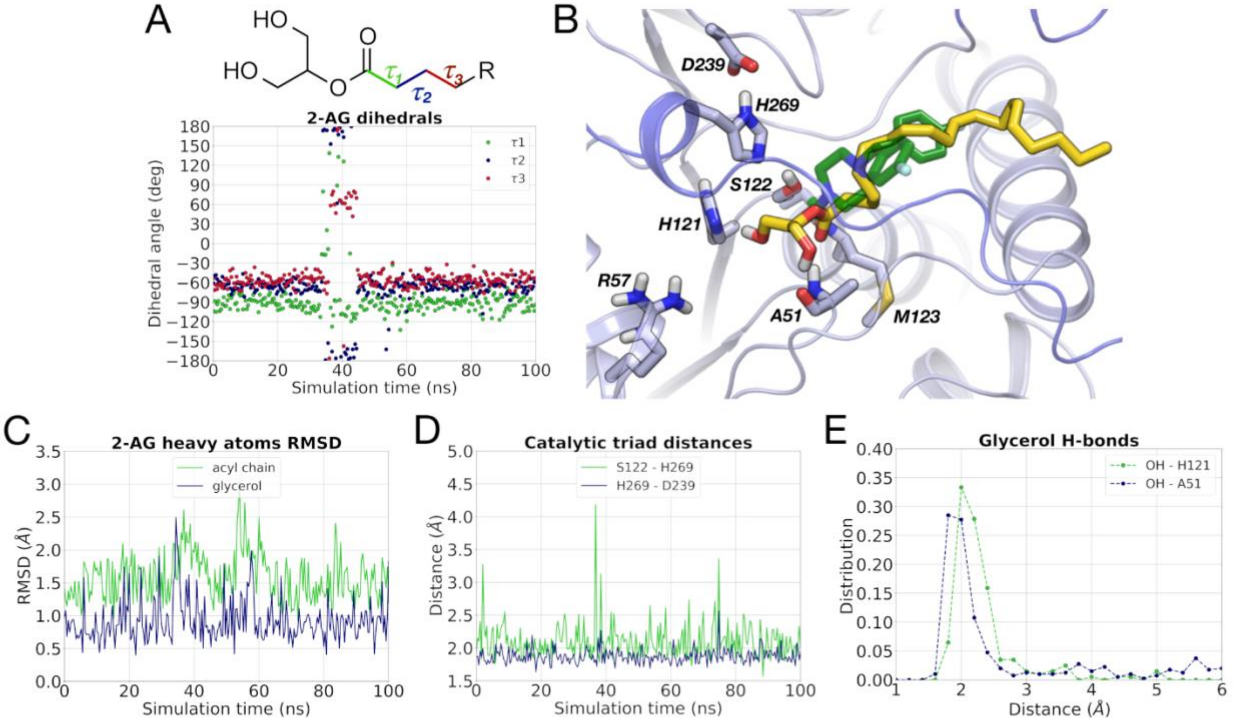

Figure S1. A) Definition and evolution of the three dihedral angles (τ1, τ2 and τ3) considered to evaluate the dynamic stability of the acyl chain of **1**. B) Superposition of a snapshot from a MD simulation of MGL–**1** (grey and yellow carbon atoms, respectively) and the X-ray of **4** (dark green carbon atoms) bound to MGL (PDB code: 3JWE). Residues involved in the recognition and hydrolysis of the substrate are represented. The secondary structure of MGL is displayed in grey cartoon, and the flexible region of the lid domain is highlighted in blue. C) Root mean square (RMSD) analysis for the heavy atoms of the acyl chain and glycerol portions of **1** during a MD simulation. D) Evolution of the hydrogen bond (H-bond) network between the catalytic triad residues of MGL–**1** complex during a MD simulation. E) Distribution of the H-bond distance of an OH group of **1** with the Nε of His121 side chain (green lines) and of the H-bond distance of the other OH group of **1** with the carbonyl oxygen of Ala51 backbone (blue lines).

Steered MD simulation for MGL acylation by 1.

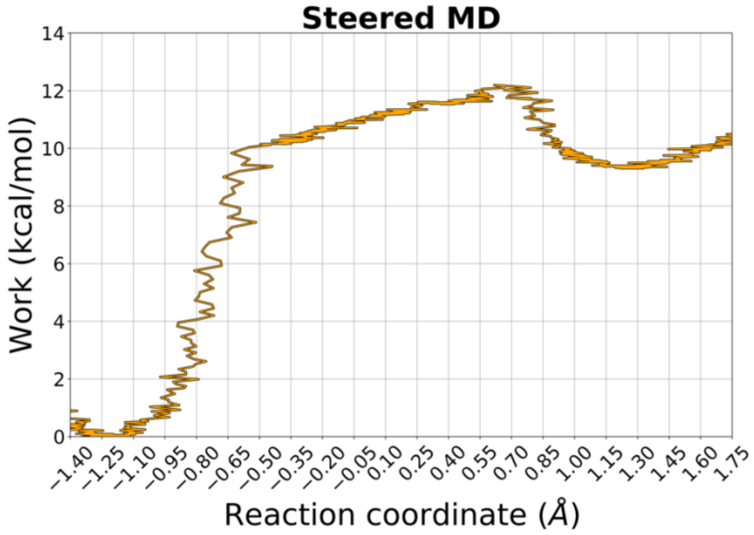

Figure S2. Work profile from a SMD simulation modeling the carbamoylation of MGL by **1**. Work profile is reported over the reaction coordinate (RC) accounting for the nucleophilic attack of Ser122 to the carbonyl carbon of the reactive ester group of **1**, and for the expulsion of glycerol as leaving group (LG).

Potential of mean force for MGL acylation by 1.

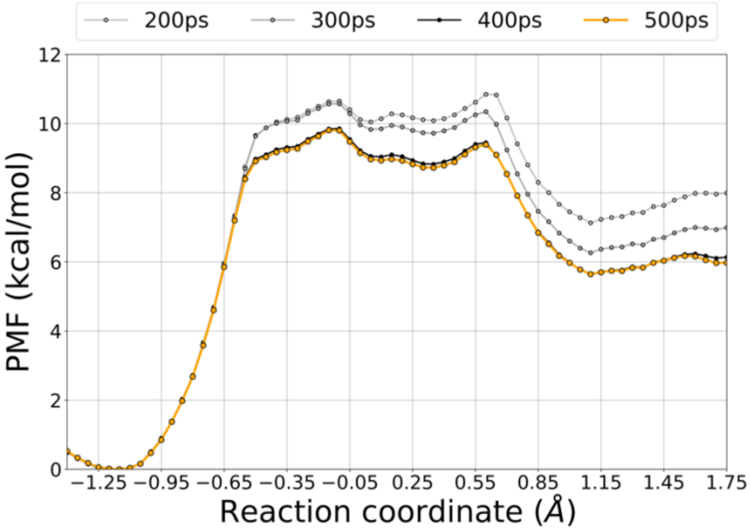

Figure S3. Evolution of the free-energy profiles at different simulation times for MGL acylation by **1**. PMF convergence is achieved after 400 ps of umbrella sampling (US) simulations, for each window.

Superposition of MGL acylated by 1 and two X-ray structures of MGL and a glycerol molecule.

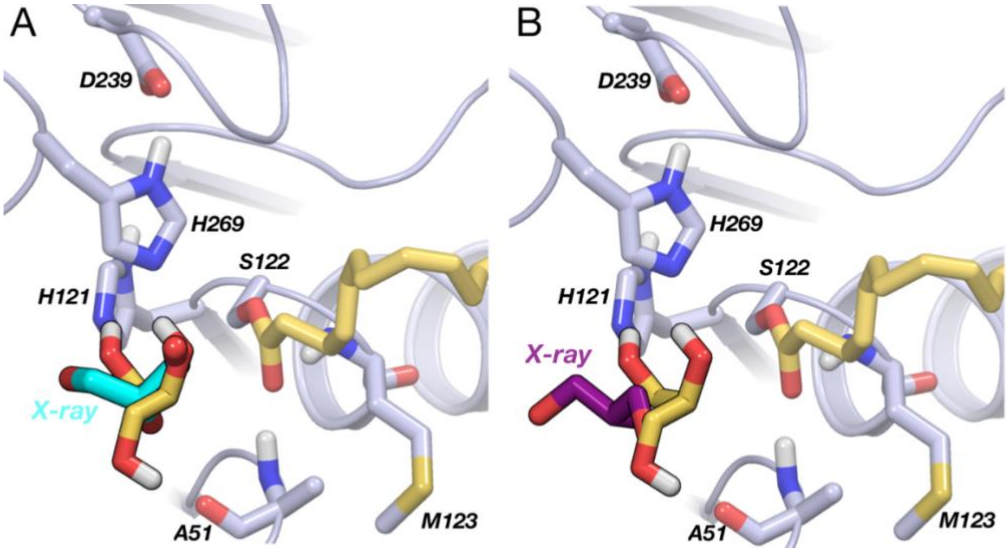

Figure S4. Superposition of MGL acylated by **1** (grey and yellow carbon atoms, respectively), representing the products of the reaction, and two X-ray structures of MGL in complex with a glycerol molecule: 6AX1 (A, cyan carbon atoms) and 3HJU (B, purple carbon atoms).

Gas-phase energy calculations on (*E*) and (*Z*)-configurations for piperazine azole urea fragments taken from compound 4–6.

Table S1. Gas phase energies (kcal·mol^{-1}) are computed at DFTB3 or M06-2X-D3/cc-PVDZ level.

Compound	Isomer	Energy (DFTB3)	Energy (M06-2X-D3)
4	(*E*)	0	0
	(*Z*)	6.56	5.28
5	(*E*)	0	0
	(*Z*)	5.93	5.57
6	(*E*)	0	0
	(*Z*)	5.52	5.33

MD simulation of MGL–4.

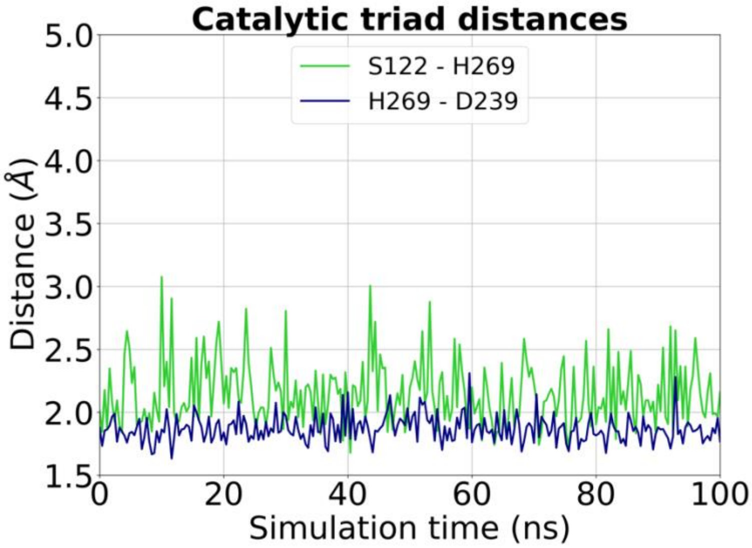

Figure S5**.** Evolution during a MD simulation of the H-bond network between the catalytic triad residues of MGL–**4** complex.

Deplanarization of azole ureas 4–6 during QM/MM MD simulations.

Table S2. Improper torsion θ and dihedral angle δ for azole ureas **4**–**6** collected from QM/MM MD simulations. Values (reported in degrees) are expressed as average ± standard error of the mean.

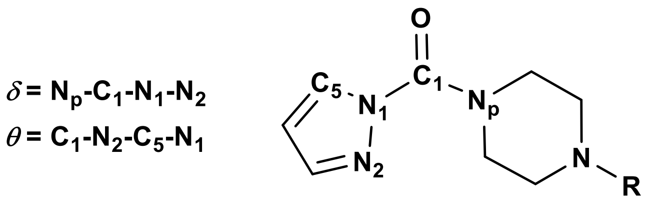

Compound	*δ*	*θ*
4	-5.8 ± 0.91	-0.89 ± 0.17
5	1.4 ± 0.6	-1.9 ± 0.14
6	5.4 ± 0.82	-0.77 ± 0.14

Steered MD simulation for MGL carbamoylation by 4.

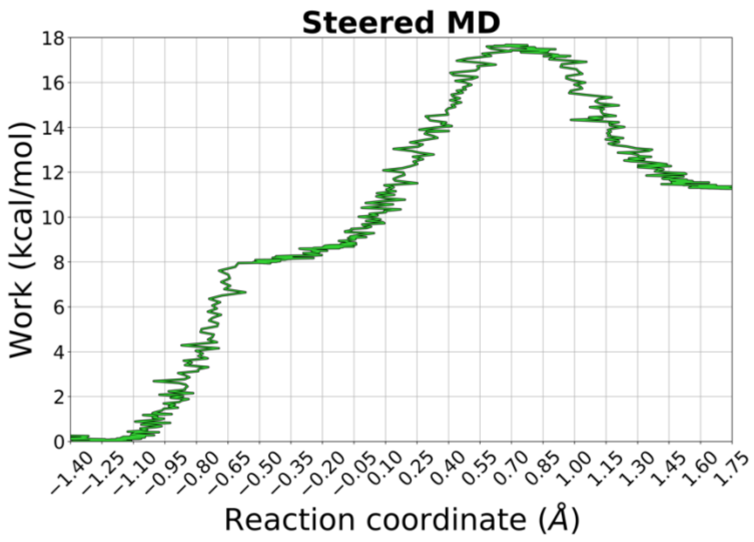

Figure S6. Work profile from a SMD simulation modeling the carbamoylation of MGL by **4**. Work profile is reported over the reaction coordinate accounting for the nucleophilic attack of Ser122 to the carbonyl carbon of the reactive urea group of **4**, and for the expulsion of the triazole ring as LG.

Potential of mean force for MGL carbamoylation by 4.

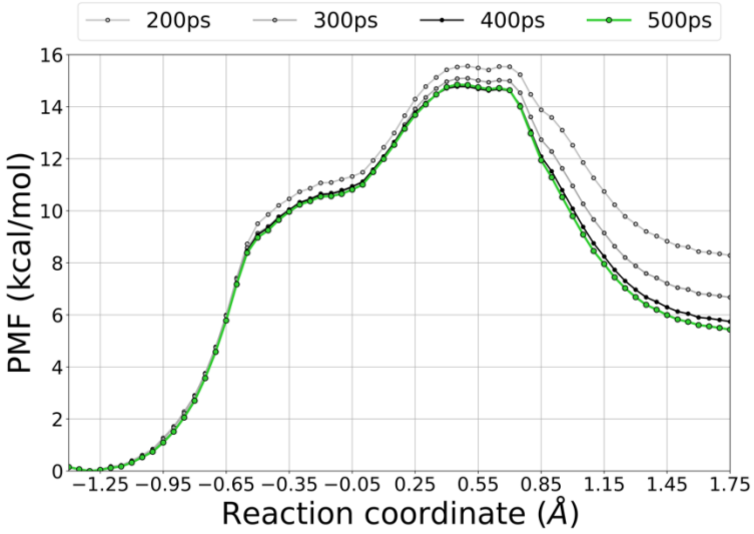

Figure S7. Evolution of the free-energy profiles at different simulation times for MGL carbamoylation by **4**. PMF convergence is achieved after 400 ps of US simulation, for each window.

Superposition of MGL carbamoylated by 4 and the X-ray structure of MGL–4.

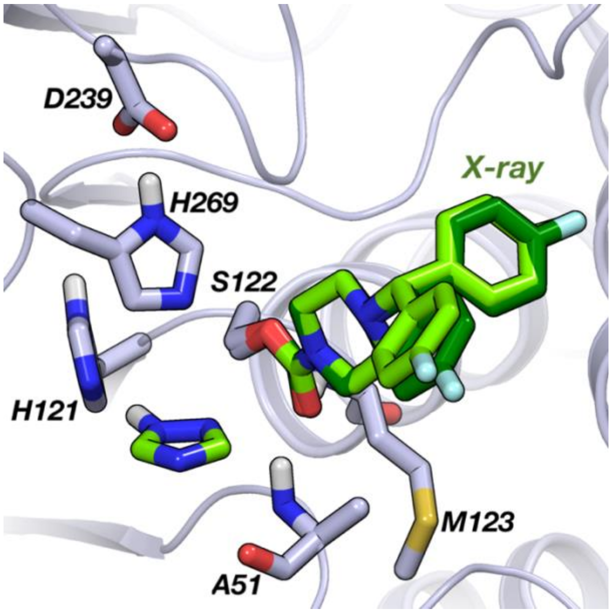

Figure S8. Superposition of MGL carbamoylated by **4** (grey and light green carbon atoms, respectively), representing the product of the reaction, and the X-ray structure of **4** (dark green carbon atoms) covalently bound to Ser122.

Docking scores for compounds 4–6.

Table S3. Docking scores in kcal·mol^{-1} obtained for compounds **4**–**6** in the (*E*)-configuration.

**Compound**	**G_{score} (kcal·mol^{-1})**
4	-6.4
5	-6.8
6	-7.6

Molecular dynamics simulations of MGL–5 and MGL–6.

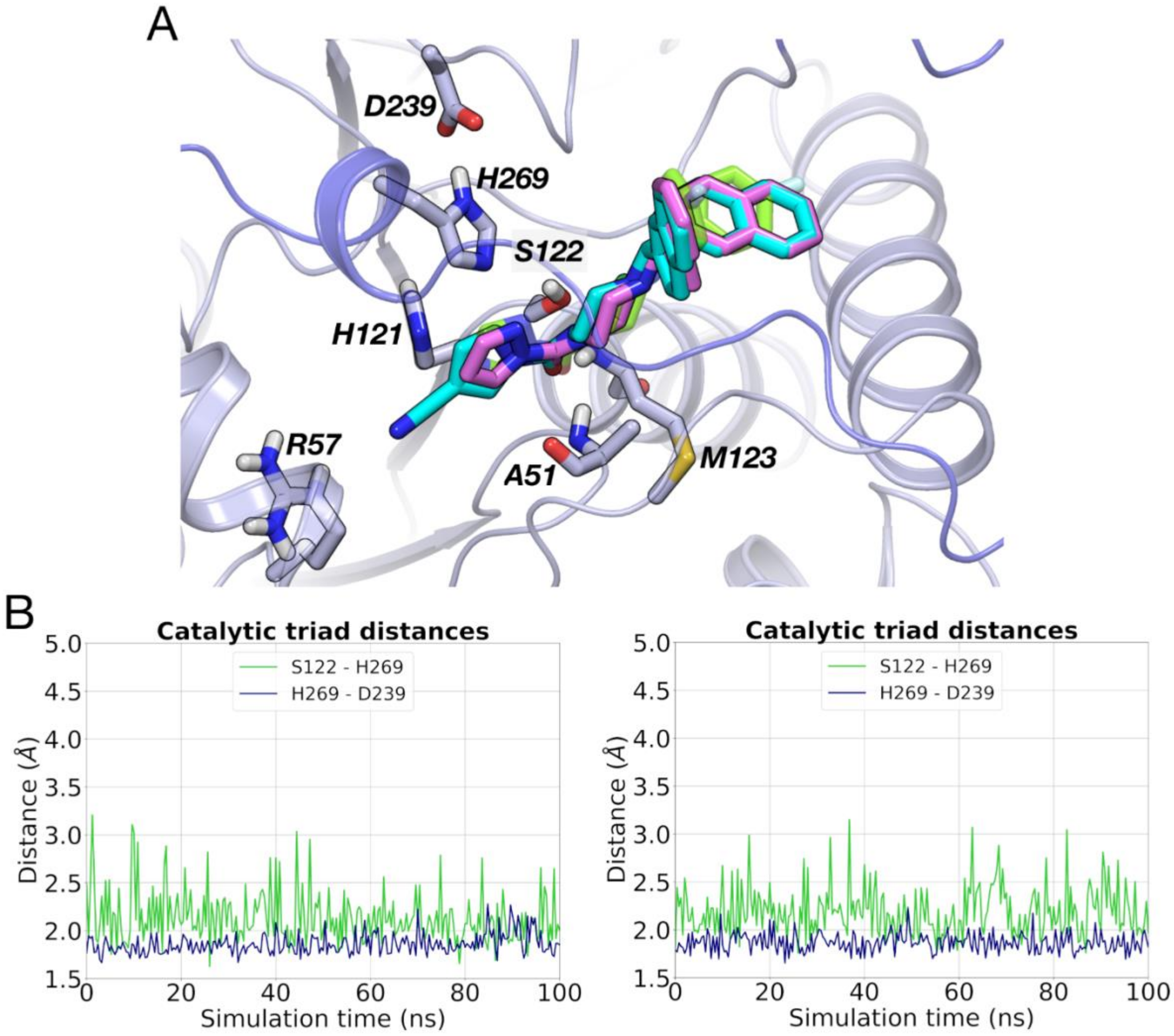

Figure S9. A) Superposition of the binding modes of **4** (light green carbon atoms in transparency) **5** (pink carbon atoms) and **6** (cyan carbon atoms) within MGL active site (grey carbon atoms). B) Evolution during a MD simulation of the H-bond network between the catalytic triad residues of MGL–**5** (left) and MGL–**6** (right) complexes.

Steered MD simulation for MGL carbamoylation by 5 and 6.

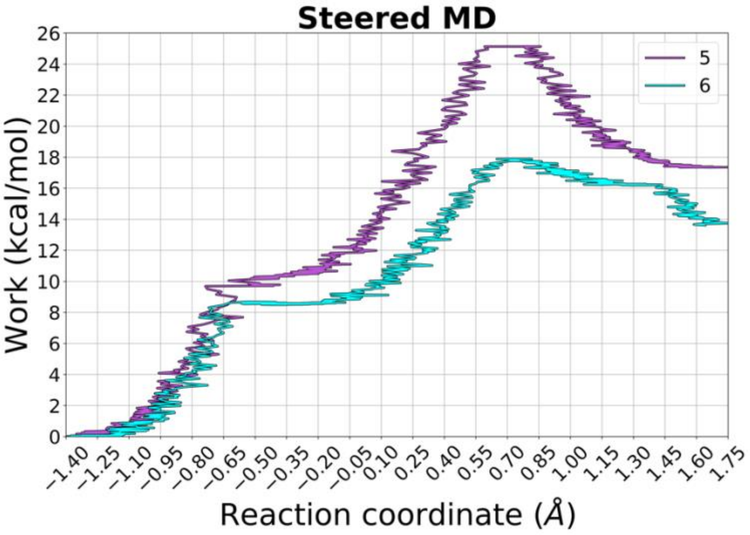

Figure S10. Work profiles from SMD simulations modeling the carbamoylation of MGL by **5** and **6**. Work profiles are reported over the reaction coordinate accounting for the nucleophilic attack of Ser122 to the carbonyl carbon of the reactive urea group of **5** and **6**, and for the expulsion of the azole ring as LG.

Potential of mean force for MGL carbamoylation by 5.

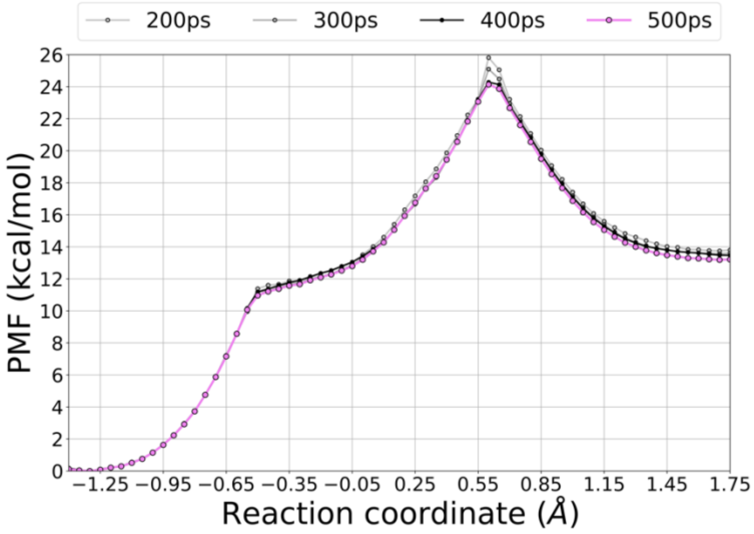

Figure S11. Evolution of the free-energy profiles at different simulation times for MGL carbamoylation by **5**. PMF convergence is achieved after 400 ps of US simulation for each window.

Potential of mean force for MGL carbamoylation by 6.

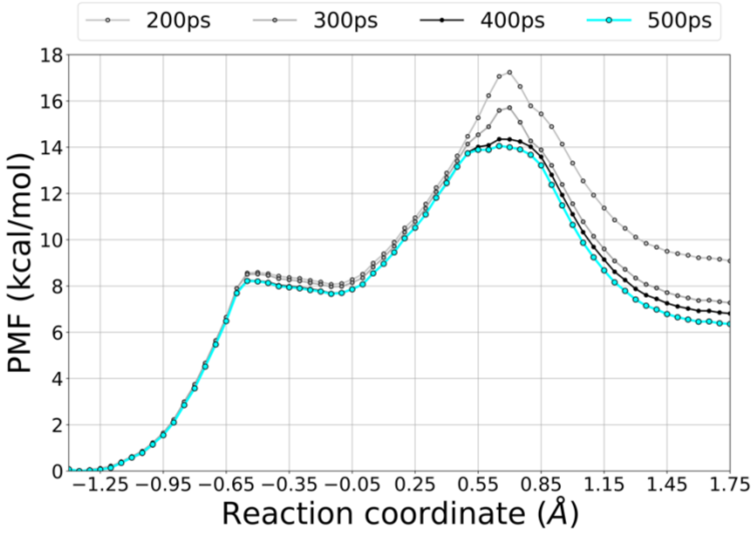

Figure S12. Evolution of the free-energy profiles at different simulation times for MGL carbamoylation by **6**. PMF convergence is achieved after 400 ps of US simulation for each window.

Polar interaction between 6 and Arg57 during US simulations.

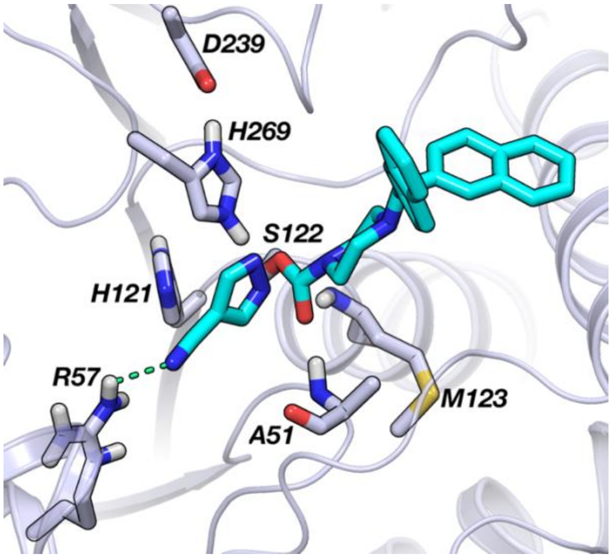

Figure S13. Representation of a snapshot from an US simulation of the transition state (TS) configuration of MGL carbamoylation by **6** (cyan carbon atoms), which forms a polar interaction with Arg57 side chain (green dashes).

Docking model of 7 within MGL binding site.

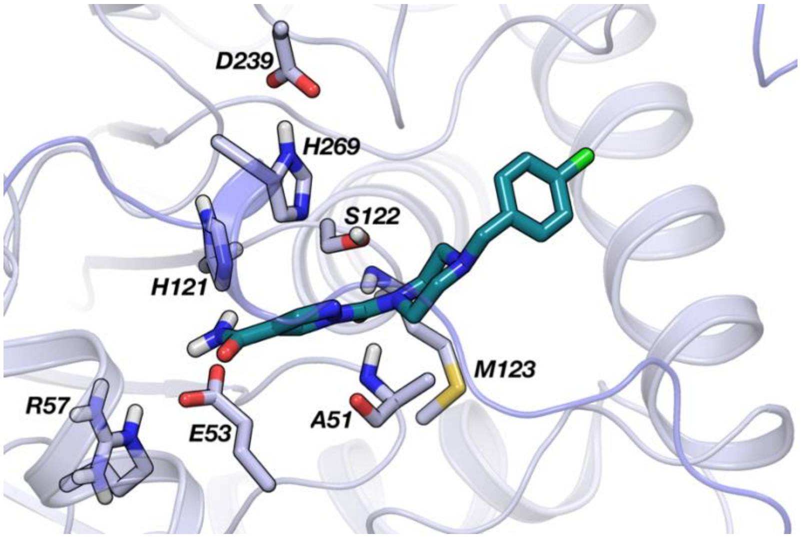

Figure S14. Binding mode of **7** (teal carbon atoms) within MGL active site (grey carbon atoms).

Steered MD simulation for MGL carbamoylation by 7.

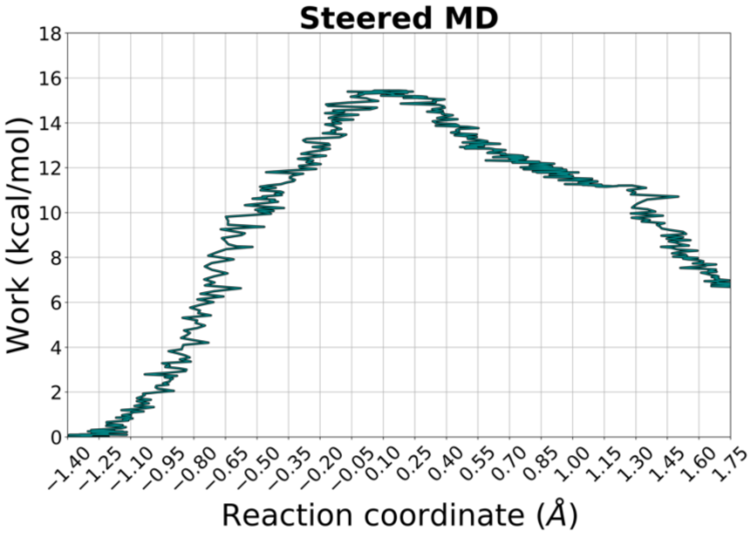

Figure S15. Work profile from a SMD simulation modeling the carbamoylation of MGL by **7**. Work profile is reported over the reaction coordinate accounting for the nucleophilic attack of Ser122 to the carbonyl carbon of the reactive urea group of **7**, and for the expulsion of the pyrazole-4-carboxamide ring as LG.

Potential of mean force for MGL carbamoylation by 7.

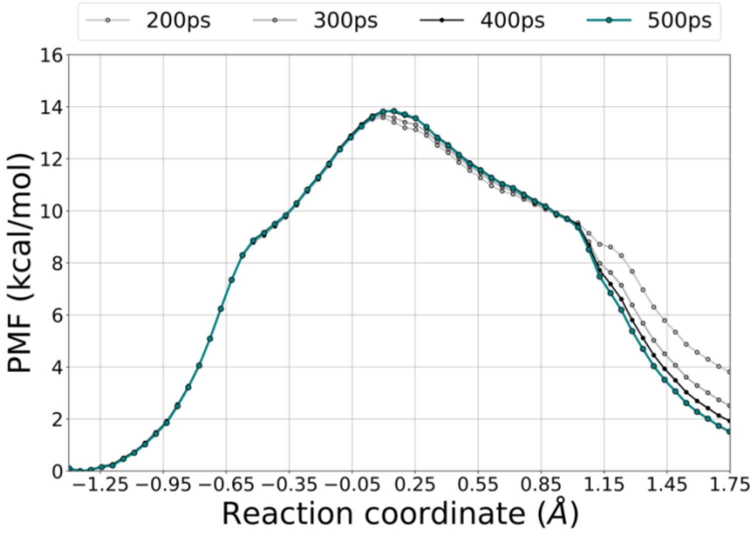

Figure S16. Evolution of the free-energy profiles at different simulation times for MGL carbamoylation by **7**. PMF convergence is achieved after 500 ps of US simulation for each window.

List of restrained residues.

In order to maintain the structure of monoglyceride lipase (MGL) close to the X-ray coordinates, and to avoid long-range conformational variations, a harmonic restraint of 5 kcal·mol^{-1} Å^{-2} was applied on the *alpha* carbon atoms of residues situated 5 Å from the active site region. This restrained region comprises all *alpha* carbon atoms of all the residues, excluding Gly50 to Asp53, Gly120 to Ile127, Ile145 to Leu148, Leu150 to Asn152, Phe209 to Val217, Leu241, Cys242, His269, Val270.

Definition of the QM region for the Michaelis complexes under study.

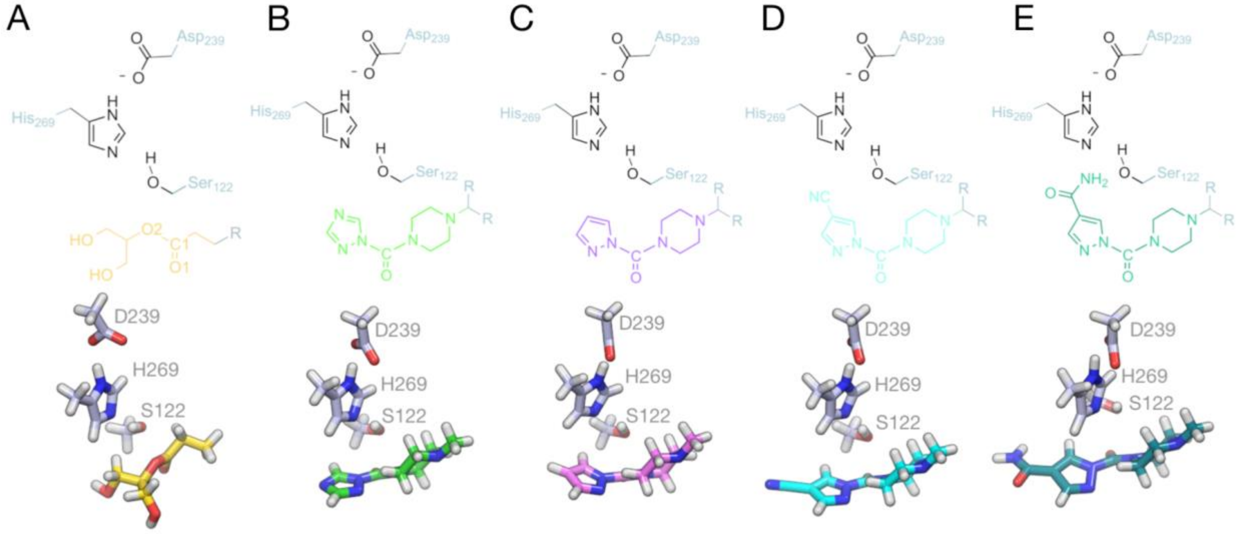

Figure S17. 2D and 3D representation of the atoms and of the link atoms included in the QM region for the Michaelis complex of MGL and **1** (A), **4** (B), **5** (C), **6** (D) and **7** (E).
